# Diverse effects of interferon alpha on the establishment and reversal of HIV latency

**DOI:** 10.1371/journal.ppat.1008151

**Published:** 2020-02-28

**Authors:** Renée M. Van der Sluis, Jennifer M. Zerbato, Jake W. Rhodes, Rachel D. Pascoe, Ajantha Solomon, Nitasha A. Kumar, Ashanti I. Dantanarayana, Surekha Tennakoon, Jérémy Dufloo, James McMahon, Judy J. Chang, Vanessa A. Evans, Paul J. Hertzog, Martin R. Jakobsen, Andrew N. Harman, Sharon R. Lewin, Paul U. Cameron

**Affiliations:** 1 The Peter Doherty Institute for Infection and Immunity, The University of Melbourne and Royal Melbourne Hospital, Melbourne, VIC, Australia; 2 Aarhus Institute of Advanced Studies, Aarhus University, Aarhus, Denmark; 3 School of Medical Sciences, Faculty of Medicine and Health, University of Sydney, Sydney, Australia; 4 Centre for Virus Research, The Westmead Institute for Medical Research, Westmead, NSW, Australia; 5 Department of Infectious Diseases, Alfred Health and Monash University, Melbourne, VIC, Australia; 6 Centre for Innate Immunity and infectious Disease, Hudson Institute of Medical Research, Clayton, VIC, Australia; 7 Dept Molecular & Translational Sciences, Monash University, Clayton, VIC, Australia; 8 Department of Biomedicin, Aarhus University, Aarhus, Denmark; Vaccine Research Center, UNITED STATES

## Abstract

HIV latency is the major barrier to a cure for people living with HIV (PLWH) on antiretroviral therapy (ART) because the virus persists in long-lived non-proliferating and proliferating latently infected CD4^+^ T cells. Latently infected CD4^+^ T cells do not express viral proteins and are therefore not visible to immune mediated clearance. Therefore, identifying interventions that can reverse latency and also enhance immune mediated clearance is of high interest. Interferons (IFNs) have multiple immune enhancing effects and can inhibit HIV replication in activated CD4^+^ T cells. However, the effects of IFNs on the establishment and reversal of HIV latency is not understood. Using an in vitro model of latency, we demonstrated that plasmacytoid dendritic cells (pDC) inhibit the establishment of HIV latency through secretion of type I IFNα, IFNβ and IFNω but not IFNε or type III IFNλ1 and IFNλ3. However, once latency was established, IFNα but no other IFNs were able to efficiently reverse latency in both an in vitro model of latency and CD4^+^ T cells collected from PLWH on suppressive ART. Binding of IFNα to its receptor expressed on primary CD4^+^ T cells did not induce activation of the canonical or non-canonical NFκB pathway but did induce phosphorylation of STAT1, 3 and 5 proteins. STAT5 has been previously demonstrated to bind to the HIV long terminal repeat and activate HIV transcription. We demonstrate diverse effects of interferons on HIV latency with type I IFNα; inhibiting the establishment of latency but also reversing HIV latency once latency is established.

## Author summary

Antiretroviral therapy (ART) cannot cure HIV or eliminate infection from long-lived and proliferating latently infected CD4^+^ T cells. Plasmacytoid dendritic cells (pDC) are major producers of interferons (IFNs), which have multiple effects on viral replication and immunity including suppression of viral expression that could favor HIV latency. Van Der Sluis et al. show that type I IFNs inhibit the establishment of HIV latency, however, once established, latency can be reversed by IFNα but not by other type I or type III IFNs. These observations demonstrate that pDC through type I IFNs are important in HIV latency and can potentially be manipulated to eliminate latent infection.

## Introduction

Antiretroviral therapy (ART) has revolutionized the treatment of HIV and has dramatically reduced mortality and morbidity. However ART is lifelong, expensive and has some long term adverse effects so there is a need to identify strategies to cure HIV or allow individuals to safely stop ART with the virus remaining undetectable, commonly referred to as remission or functional cure, reviewed by [1]. The major barrier to a cure for HIV is the persistence of latent infection in long-lived and proliferating CD4^+^ T cells [2–5]. Multiple factors contribute to both the establishment and maintenance of HIV latency, including the host chromatin environment at the site of HIV integration and reduced transcription factors that are required for virus production (reviewed by [1]).

Type I interferons (IFNs) and the more recently identified type III IFNs function as the first line of defense against virus infection [6, 7]. IFNs can induce inflammation, activate specific immune cells including natural killer (NK) cells, CD8^+^ T cells and macrophages, and prime antigen-specific responses. IFNs also have a potent direct antiviral effect and can inhibit productive HIV replication by increasing the expression of multiple cellular restriction factors that inhibit steps in the HIV life cycle prior to and following HIV integration [8–11].

In the context of latent HIV infection, IFNs could have multiple effects on both the establishment and maintenance of latency. IFNs could induce expression of restriction factors active prior to HIV integration, such as tripartite motif-containing (TRIM) 5α [11]. Alternatively, once virus integration is established, IFNs could induce expression of restriction factors that are active post-integration which would be important in maintaining latency, such as TRIM19 or TRIM22 [12–14], which can repress virus transcription.

The administration of IFNs and the blockade of IFN *in vivo*, has highlighted diverse outcomes on HIV latency. Several studies have demonstrated that type I IFN and IFN stimulated genes (ISG) may impact HIV latency in people living with HIV (PLWH) on ART. The administration of IFN to PLWH on ART resulted in a decrease in integrated HIV DNA, suggesting a decrease in reservoir size through an unclear mechanism [15–19], however these findings were not replicated in simian immunodeficiency virus (SIV) infected non-human primates (NHP) on suppressive ART [20]. In contrast to these observations, IFN may potentially enhance HIV persistence on ART, because in an HIV-infected humanized mouse on suppressive ART, blocking type I interferon signaling resulted in a reduction in the viral reservoir and a delay in time to viral rebound following cessation of ART [21].

The administration of toll-like receptor (TLR) 7 agonists, a potent stimulator of type I IFN, to SIV-infected NHP on suppressive ART initiated in chronic SIV infection, resulted in marked increases in expression of the activation marker CD69 on CD4^+^ and CD8^+^ T cells and concurrent increases in plasma SIV RNA consistent with latency reversal [22]. Consistent with these findings, treatment of peripheral blood mononuclear cells (PBMC) isolated from PLWH with a TLR7 agonist *ex vivo*, resulted in enhanced HIV expression, consistent with latency reversal, and further *in vitro* work demonstrated that the effect of the TLR7 agonist was mediated by plasmacytoid dendritic cell (pDC)-secreted IFNalpha (IFNα) [23]. However, these findings were not replicated in three subsequent studies of TLR7 agonists administered to non-human primates, either early following infection or after prolonged ART, where no change in plasma SIV RNA was observed [24–26]. Treatment of PLWH on ART with the TLR9 agonist MGN1703 was associated with increases in plasma HIV RNA consistent with latency reversal [27] and increased expression of type I IFN and the restriction factors MX1, ISG15, IFITM1, MX2 and TRIM22 in the gut [28]. However, a recent clinical trial of the TLR7 agonist GS-9620 in PLWH on ART showed no increase in plasma HIV RNA. Collectively, these studies suggest that IFN (and agents that induce IFN) may mediate variable effects on HIV latency–which may be a direct virological or an indirect immune-mediated effect and may also be dependent on the frequency and transcriptional activity of latently infected cells in the participant [26].

pDC are a major source of type I IFN production in response to virus infection via sensing of viral products such as single stranded RNA by TLR7 or unmethylated DNA molecules by TLR9 (reviewed in [29]. We have previously reported that myeloid dendritic cells (mDC) and monocytes induce the establishment of latent HIV infection in non-proliferating and proliferating CD4^+^ T cells within an *in vitro* co-culture model [30–32]. In contrast, pDC did not facilitate the establishment of latent HIV infection. Given that pDC produce abundant type I IFN [33] and type III IFNs [34], here we investigated how pDC modulate HIV latency in CD4^+^ T cells and studied the effect of individual type I and III IFNs on the establishment of latent infection as well as the effect of different IFNs on latently infected cells. Using this *in vitro* model, we demonstrate that type I IFNα, IFNbeta (IFNβ) and IFNomega (IFNω) all inhibit HIV, but IFNα was more potent than IFNβ and IFNω pre-integration. Once latency was established, IFNα was able to induce virus expression from latent HIV consistent with latency reversal, potentially mediated by phosphorylation of STAT5 but not via the activation of the NFκB signaling pathway. These observations demonstrate the significant but diverse direct effects of IFNs on HIV latency establishment and reversal.

## Results

### Plasmacytoid DC inhibit HIV latency via type I IFNs

**Fig 1:**
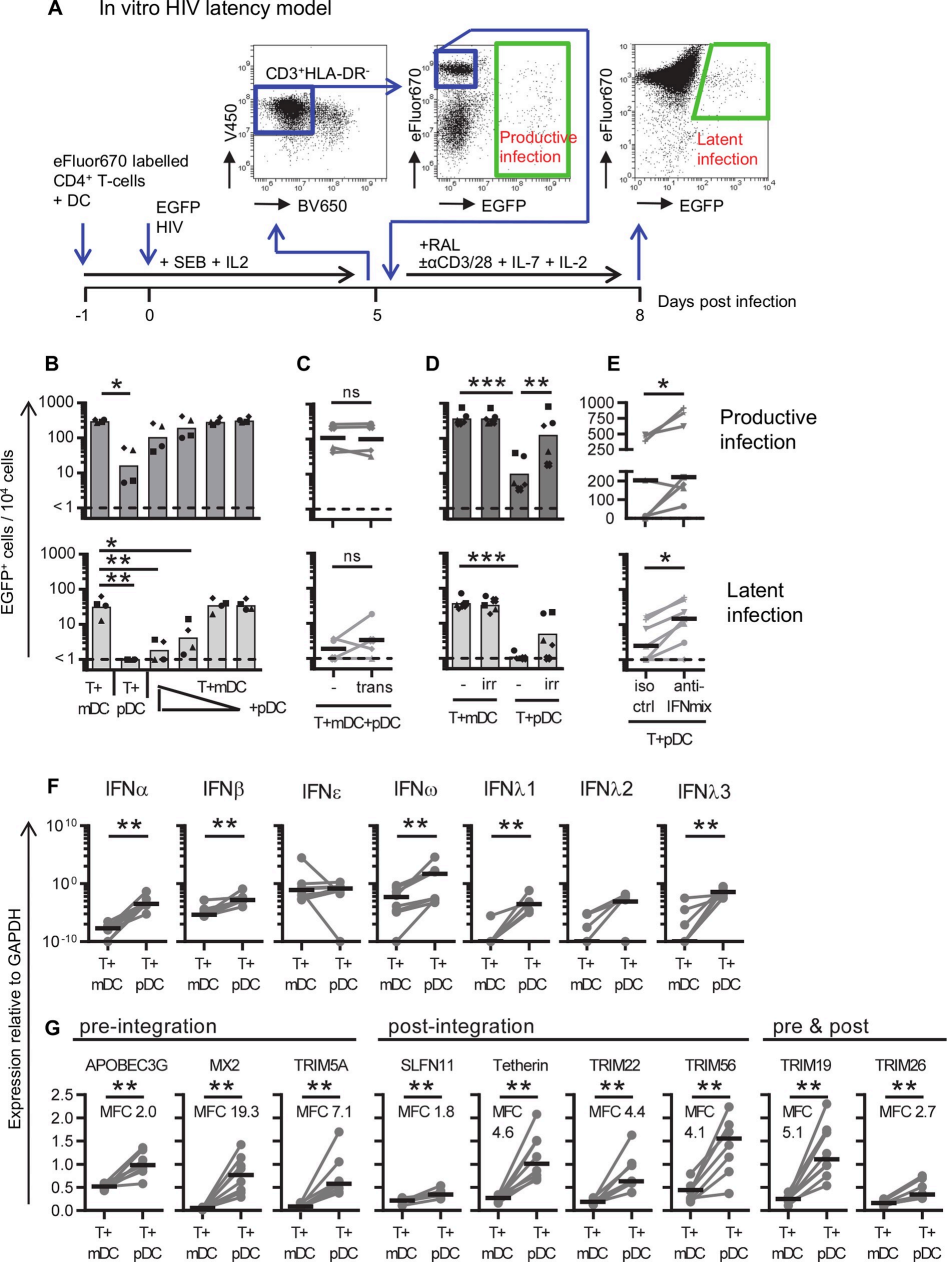
pDC-induced inhibition of HIV latency. **A:** Schematic of the *in vitro* DC-T cell HIV latency model. Resting CD4^+^ T cells were negatively selected using magnetic cell sorting, stained with the proliferation dye eFluor670 and cultured with and without syngeneic sorted DC subsets (DC:T cell ratio of 1:10) for 24 hrs. in the presence of staphylococcal enterotoxin B (SEB) and IL-2. Cells were infected with CCR5-using full-length nef-competent EGFP-reporter virus (EGFP HIV). At day 5 post-infection, EGFP^+^ cells were measured by flow cytometry and used as a measure for productive infection. The CD3^+^HLA-DR^-^ non-productively infected (EGFP^-^), non-proliferating (eFluor670^HI^) CD4^+^ T cells were sorted and cultured with an HIV integrase inhibitor (raltegravir; RAL) in the presence or absence of activation stimuli (anti-CD3/CD28+IL-7+IL-2) for 72 hrs. After stimulation, EGFP expression was measured by flow cytometry and latent infection quantified as the number of EGFP^+^ cells in the stimulated culture after subtracting the number of EGFP^+^ cells in the unstimulated culture (background). Resting CD4^+^ T cells were co-cultured with **B:** mDC, pDC, or both at different ratios of mDC:pDC (1:1, 10:1, 100:1 and 1,000:1, n = 4), **C:** mDC and pDC were co-cultured at a 1:1 ratio, in the absence (-) or presence of a transwell (trans, n = 4), **D:** T cells were co-cultured with mDC or pDC that were irradiated (irr) or not (-, n = 5, DC:T cell ratio of 1:10), **E:** T cells were cultured with pDC in the presence of antibodies blocking IFNAR and neutralizing soluble IFNα and IFNβ (anti-IFNmix) or an isotype control (iso ctrl, n = 7, DC:T cell ratio of 1:10). Productive (dark grey, top panels) and latent (light grey, bottom panels) infection in non-proliferating cells was quantified and shown in panels B-E. **F,G:** Resting CD4^+^ T cells were co-cultured with pDC or mDC (DC:T cell ratio of 1:10), infected with EGFP HIV, harvested at 1 day post-infection, RNA was extracted and reverse transcribed into cDNA for qPCR detection of **F:** type I IFNs (IFNα, IFNβ, IFNε, IFNω) and type III IFNs (IFNλ1/IL-29, IFNλ2/IL-28A, IFNλ3/IL-28B, n = 8) or **G:** virus restriction factors that inhibit HIV replication prior to integration, post-integration, or both (n = 8, MFC = mean fold change). Values were normalized to mRNA of the house keeping gene GAPDH. Columns or lines represent mean values (n≤5) or median values (n>5). Symbols and dots represent individual donors. *p<0.05, **p<0.01, ***p<0.001, ns = not significant, as determined by paired student T test (n≤5) on log-transformed data or Wilcoxon matched pairs signed rank test (n>5).

We have previously reported that HIV infection of resting CD4^+^ T cells *in vitro*, in the presence of mDCs and monocytes but not pDCs, enhanced the establishment of latent infection in CD4^+^ T cells [30–32]. In this *in vitro* model of HIV latency (Fig 1A), resting CD4^+^ T cells were stained with the proliferation dye eFluor670 and cultured with and without syngeneic sorted DC subsets (DC:T cell ratio of 1:10) for 24 hrs in the presence of staphylococcal enterotoxin B (SEB) and interleukin (IL)-2. Cells were then infected with CCR5-using full-length Nef-competent virus expressing enhanced green fluorescent protein (EGFP) under the control of the HIV long terminal repeat (LTR) (EGFP HIV). At day 5 post-infection, EGFP^+^ cells were quantified by flow cytometry and used as a measure for productive infection. Subsequently, the CD3^+^HLA-DR^-^ non-productively infected (EGFP^-^), non-proliferating (eFluor670^HI^) CD4^+^ T cells were sorted and cultured with an HIV integrase inhibitor (raltegravir; RAL) in the presence or absence of an activation stimulus (anti-CD3/CD28+IL-7+IL-2) for 72 hrs. After stimulation, EGFP expression was measured by flow cytometry and latent infection quantified as the number of EGFP^+^ cells in the stimulated culture minus the number of EGFP^+^ cells in the unstimulated culture (background).

To investigate if pDCs prevent latent infection via a dominant negative effect, we infected CD4^+^ T cells in the presence of mDCs and the pDCs were added back to the CD4^+^ T cell—mDC co-cultures at decreasing ratios of pDC to mDC (ranging from 1:1 to 1:1000). As previously reported, mDCs facilitated higher levels of productive infection in non-proliferating CD4^+^ T cells than pDCs (mean number of infected cells 307 and 27 EGFP^+^ cells/10^4^ cells, respectively, p = 0.019, Fig 1B top panel). Latent infection was also significantly higher in CD4^+^ T cells cultured with mDCs compared to pDCs (mean number of latently infected cells 37 and 1 EGFP^+^ cells/10^4^ cells, respectively, p = 0.0019, Fig 1B bottom panel). Adding pDCs to co-cultures of CD4^+^ T cells and mDCs resulted in a dose-dependent reduction of both productive and latent infection, indicating a pDC dominant negative effect (Fig 1B).

To investigate if pDCs inhibited the establishment of HIV latency in non-proliferating CD4^+^ T cells, by soluble factors or cell-cell interaction, we used transwell cultures. The CD4^+^ T cells were cultured with mDCs in the bottom compartment and pDCs added to the top compartment, thereby preventing direct contact between pDCs and CD4^+^ T cells, yet allowing pDC-secreted soluble factors to be available to the CD4^+^ T cell and mDC co-cultures. The number of productively and latently infected cells did not change in the presence of a transwell (Fig 1C), indicating that the effect of pDC was mediated by soluble factors. To confirm this finding, resting CD4^+^ T cells were co-cultured with irradiated mDCs and pDCs. Irradiation of DCs allows for cell-cell interaction while inhibiting some DC-secreted soluble factors [35]. Co-culture of resting CD4^+^ T cells with irradiated mDCs did not significantly change the level of productive and latent infection (Fig 1D), indicating that mDCs facilitate infection via a cell-cell interaction rather than via mDC-secreted soluble factors. When CD4^+^ T cells were co-cultured with irradiated pDCs there was an increase in the frequency of productively infected cells compared to non-irradiated pDCs (mean 17 and 272 EGFP^+^/10^4^ cells respectively, mean fold change (MFC) 18, p = 0.003) and latently infected cells (mean 1 and 9 EGFP^+^/10^4^ cells respectively, MFC 8, p = 0.051), (Fig 1D). Together these results indicate that pDCs secrete soluble factors that inhibit productive and latent infection.

Given that pDC produce abundant type I interferons (IFNs) [33], we sought to investigate if blocking type I IFN signaling would enhance the establishment of latent infection, rather than measuring IFN production post-irradiation. We have previously performed these experiments by blocking IFNα and found there to be no effect [30]. To extend the inhibition beyond IFNα, resting CD4^+^ T cells were co-cultured with pDCs in the presence of antibodies blocking the type I IFN receptor (IFNAR) and antibodies that neutralize IFNα and IFNβ, hereafter designated anti-IFN antibodies, or control antibodies of the same isotype (S1A Fig). Productive and latent infection significantly increased when resting CD4^+^ T cells were co-cultured with pDCs in the presence of anti-IFN antibodies compared to the isotype control (MFC 22, p = 0.03 and MFC 5, p = 0.03, respectively) (Fig 1E). These results indicate that pDC-secreted type I IFNs inhibit productive and latent infection of resting CD4^+^ T cells.

### Type I and III interferons are expressed at higher levels in T cell + pDC than in T cell + mDC co-cultures

To investigate if other IFNs were present in the co-cultures of CD4^+^ T cells with either mDCs or pDCs, type I and III IFN gene expression levels were measured by qPCR 24 hrs post-infection. Compared to CD4^+^ T cell + mDC co-cultures, the CD4^+^ T cell + pDC co-cultures had significantly higher levels of type I IFNα (MFC 3.4x10^6^, p = 0.008), IFNβ (MFC 3.5x10^4^, p = 0.008) and IFNω, (MFC 2.6x10^6^, p = 0.008), and type III IFNlambda1 (IFNλ1/IL-29, MFC 8.5x10^7^, p = 0.008), and IFNλ3 (IL-28B, MFC 8.8x10^8^, p = 0.008), as expected (Fig 1F). There was no difference in the expression of type I IFNepsilon (IFNε) or type III IFNλ2 between mDCs and pDCs (Fig 1F). In contrast, HIV infection of co-cultures of CD4^+^ T cells and pDCs enhanced mRNA expression of IFNα (MFC 120, p = 0.03), IFNβ (MFC 62, p = 0.03), IFNω (MFC 1700, p = 0.04) and IFNλ2 (MFC 420, p = 0.04) (S1B Fig). HIV infection did not significantly increase IFNε, IFNλ1 or IFNλ3 mRNA expression (S1B Fig). These results show that pDCs express higher levels of type I IFNα, IFNβ and IFNω, and type III IFNλ1 and IFNλ3 genes compared to mDCs.

### Virus restriction factor expression is increased in the presence of pDCs

Interferon stimulated genes (ISGs) encode effector proteins that protect against viral infection and regulate innate and adaptive immune cells [9]. Some ISGs are HIV restriction and virus inhibition factors that modulate productive infection in activated CD4^+^ T cells [10, 13, 14, 36–41]. To investigate whether known HIV restriction factors were expressed in the *in vitro* culture system used here, mRNA was extracted from resting CD4^+^ T cells co-cultured with mDCs or pDCs and restriction factors were quantified by qPCR. All HIV restriction factors measured were detected at significantly higher levels in CD4^+^ T cells cultured in the presence of pDCs compared to mDCs (Fig 1G).

### Type I IFNs inhibit mDC-induced latent infection in non-proliferating T cells

**Fig 2:**
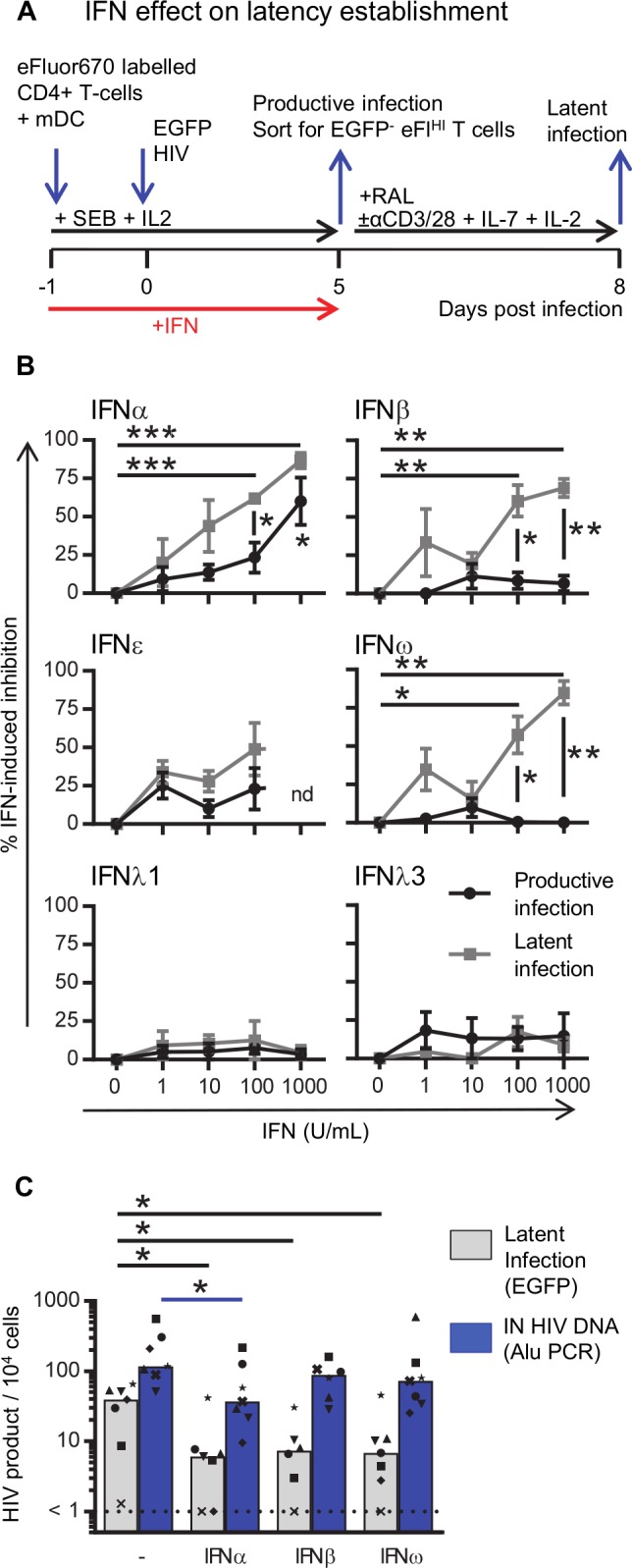
IFN-induced inhibition of productive infection and establishment of latent infection. **A:** Experimental design to determine the effect of IFN on productive and latent infection. **B:** Resting CD4^+^ T cells were co-cultured with mDC in the absence or presence of IFN (1–1000 U/mL, as indicated) and the IFN-induced inhibition was calculated for productive (black circles) and latent (grey squares) infection. **C:** Resting CD4^+^ T cells were co-cultured with mDC in the absence and presence of 100 U/mL IFNα, IFNβ or IFNω. Cells were infected and sorted as described in **A** except that part of the sorted EGFP^-^eFluor^HI^ population was lysed, DNA extracted and the number of HIV integrated copies was quantified using the Alu PCR and normalized to the housekeeping gene CCR5. The remainder of the sorted cells were cultured to quantify latent infection. Lines (**B**) indicate mean values ±SEM (n = 3–4). Columns (**C**) represent median values and dots represent individual donors with each donor represented by a specific symbol (n = 6–7 donors). *p<0.05, **p<0.01, ***p<0.001, as determined by paired student T test (n = 3–4) or Wilcoxon matched pairs signed rank test (n = 6–7), nd = not done.

In our *in vitro* model, different type I IFNs were measured by qPCR in the co-cultures of CD4^+^ T cells and pDCs, and IFN-blockade inhibited the effects of pDCs on latency (see Fig 1E). We therefore added each IFN individually to co-cultures of CD4^+^ T cells with mDC at increasing concentrations (1–1000 U/mL, Fig 2A). Addition of 1000 U/mL universal IFNα did not significantly decrease the number of productively infected cells (mean of 281 and 104 EGFP^+^ cells, p = 0.08, n = 4, S2 Fig), but there was a relative mean inhibition of 60% (range 29–89%, p = 0.03, n = 4, Fig 2B). The addition of 100 U/mL and 1000 U/mL of IFNα led to a decline in latent infection from a mean of 30 to 11 and 3 EGFP^+^ cells respectively (p = 0.002 and 0.012) and a relative mean (range) inhibition of 62 (58–71) and 87 (74–95)% respectively (p = 0.0003 and p = 0.0004).

We previously reported that in the DC-T cell latency model, the level of latent infection did not correlate with the level of productive infection [31]. The effect of IFNα on latent infection was also not a direct result of the effect of IFNα on productive infection, for there was a greater reduction of latent compared to productive infection, which was statistically significant with IFNα >100 U/ml (Fig 2B). These results suggest that IFNα may inhibit HIV at different steps of the viral replication cycle in latently and productively infected cells, consistent with the reported literature [11].

To investigate the effect of other IFNs, experiments were repeated with type I IFNβ, IFNω, and type III IFNλ1 and IFNλ3, and type I IFNε. We included IFNε because it is unique among IFNs as, in mice, the expression is regulated hormonally instead of induced by viral infection [42]. However, in human cells expression can be induced by Sendai virus and herpes simplex virus type-2 [43]. Additionally, IFNε can inhibit HIV replication in cell lines and activated peripheral CD4^+^ T cells [44]. Addition of type I IFNβ and IFNω (100 U/mL) did not affect productive infection in DC-T cell cultures but did decrease the frequency of latently infected CD4^+^ T cells with a relative mean (range) inhibition of 61 (42–89)% and 57 (40–80)% (p = 0.01, n = 4 and p = 0.04, n = 3), respectively (Fig 2B and S2 Fig). Neither type III IFNλ1 nor IFNλ3 inhibited productive or latent infection in this co-culture system. Therefore, IFNα, IFNβ and IFNω all inhibit the establishment of latent infection while only IFNα inhibited productive infection. Together, these data suggest that inhibition of productive infection following the addition of pDCs is most efficiently mediated by IFNα and that the inhibition of latent infection is likely due to other pDC-secreted type I IFNs, including IFNα, IFNβ and IFNω.

To test whether type I IFNα, IFNβ and IFNω inhibited HIV prior to integration, we also quantified integrated virus using qPCR, as previously described [45]. In CD4^+^ T cells co-cultured with mDCs infected with HIV in the absence and presence of IFNα, there was a significant reduction in the frequency of inducible EGFP (mean EGFP^+^/10^4^ cells 35 and 10, p = 0.016; a relative mean (range) inhibition of 63(22–97)%) and mean integrated (IN) HIV DNA (copies/10^4^ cells 207 and 71, p = 0.016; a relative mean (range) inhibition of 65 (51–95)%), n = 7) (Fig 2C). The addition of IFNβ and IFNω reduced inducible EGFP expression compared to IFNα (relative mean (range) percentage inhibition: IFNβ 64 (22–85)%, p = 0.03; IFNω 62 (22–93)%, p = 0.016) but there was no statistically significant difference in the effect on integrated DNA relative to IFNα (relative mean (range) percentage inhibition IN HIV DNA: IFNβ 46 (0–68)%, p = 0,06, n = 6; IFNω 48 (0–88)%, p = 0,3, n = 7). These results indicate that following HIV infection of non-proliferating CD4^+^ T cells, IFNα inhibits HIV replication prior to integration and does this more potently than IFNβ and IFNω.

### IFNα can induce HIV virus expression *in vitro* and *ex vivo*

**Fig 3:**
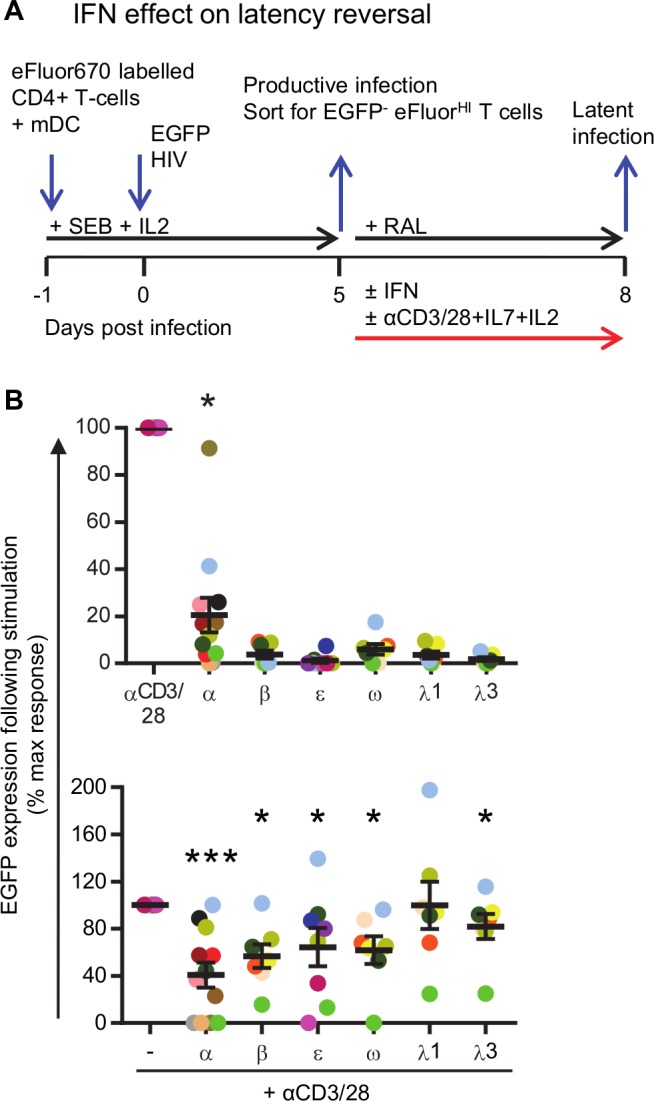
IFNα but not other type I or III IFNs induce expression of HIV. **A:** Experimental design to determine the effect of IFN on reversal of latent HIV. Resting CD4^+^ T cells were co-cultured with mDC, infected with EGFP-HIV and day 5 post-infection sorted for CD3^+^HLA-DR^-^eFluor^hi^EGFP^-^. Sorted cells were cultured in the presence of an HIV integrase inhibitor (RAL) and either left untreated, cultured with 100 U/mL IFN, activated with anti-CD3/CD28+IL-7+IL-2 or activated with anti-CD3/CD28+IL-7+IL-2 in the presence of 100 U/mL IFN **B:** After 3 days, the cells were harvested and EGP expression quantified by flow cytometry. To calculate the IFN-induced EGFP expression, EGFP expression in unstimulated conditions (background EGFP) was subtracted from EGFP in stimulated conditions. The IFN-induced EGFP expression is then shown as a percentage of maximal stimulation i.e. following stimulation with anti-CD3/CD28+IL-7+IL-2 minus background EGFP expression. Black lines indicate median values and each circle represents an individual donor (n = 7–12 donors). Statistical comparisons were made between IFN treated and untreated samples (top panel) or IFN+anti-CD3/CD28+IL-7+IL-2 and anti-CD3/CD28+IL-7+IL-2 (bottom panel). *p<0.05, ***p<0.001, ns = not significant as determined with Wilcoxon matched pairs signed rank test.

Given the clear effects of IFNα, IFNβ and IFNω in reducing the establishment of HIV latency *in vitro*, we next determined the effects of each IFN on latency reversal. Resting CD4^+^ T cells were co-cultured with mDCs, infected with HIV and sorted for CD3^+^HLA-DR^-^EGFP^-^ non-proliferating CD4^+^ T cells. The sorted cells were subsequently cultured with an integrase inhibitor, preventing new rounds of virus infection, and treated with 100 U/mL of IFN (α, β, ε, ω, λ1 or λ3) in the presence or absence of anti-CD3/CD28+IL-7+IL-2 (Fig 3A). As observed in previous experiments, anti-CD3/CD28+IL-7+IL-2 enhanced virus expression as measured by EGFP (S3 Fig). Surprisingly, treatment of latently infected CD4^+^ T cells with IFNα alone induced virus expression in 11 out of 12 donors (S3 Fig). The mean (range) effect of IFNα on HIV latency reversal was measured by quantifying EGFP expression following stimulation and expressing this value as a percentage of maximal stimulation with anti-CD3/CD28+IL-7+IL-2 which, had a mean (range) value of 21 (0–91)%, (Fig 3B). When IFNα was combined with anti-CD3/CD28+IL-7+IL-2, virus expression was inhibited by 59% compared to anti-CD3/CD28+IL-7+IL-2 without IFNα (%max stim 41(0–100)). None of the other IFNs increased EGFP expression from the latently infected cells (Fig 3B, S3 Fig) and all IFNs except IFNλ1, inhibited virus expression when combined with anti-CD3/CD28+IL-7+IL-2 compared to anti-CD3/CD28+IL-7+IL-2 without IFN (Fig 3B, S3 Fig). Compared to the maximal EGFP expression induced by anti-CD3/CD28+IL-7+IL-2 stimulation, IFNβ inhibited EGFP expression by 43%, IFNε by 36%, IFNω by 38%, IFNλ1 by 0% and IFNλ3 by 18%.

**Fig 4:**
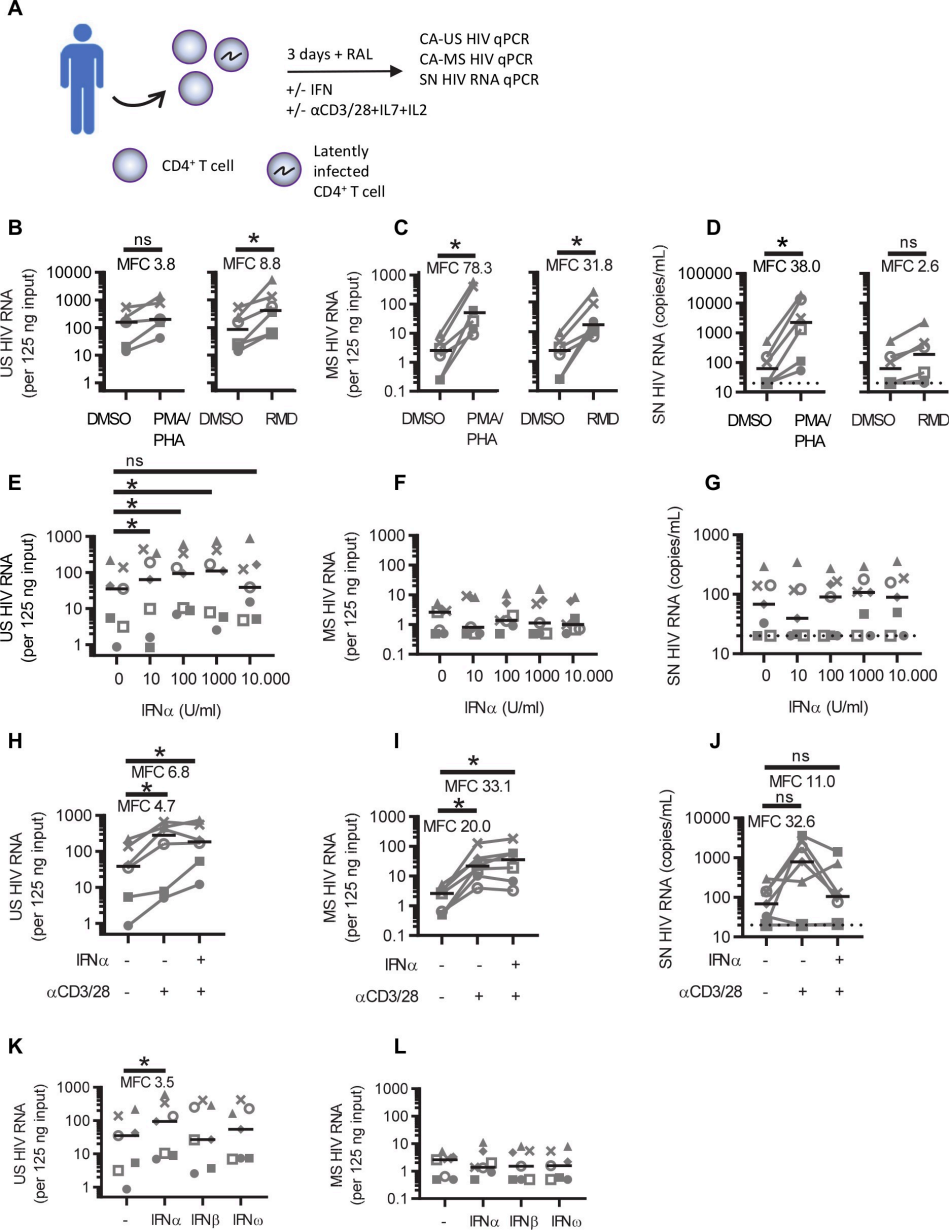
IFNα induces expression of unspliced HIV RNA *ex vivo*. **A:** Total CD4^+^ T cells were isolated from PBMC obtained via leukapheresis from people living with HIV (PLWH) on ART and stimulated in the presence of the HIV integrase inhibitor raltegravir (RAL). After 3 days the cells were harvested, RNA was isolated and reverse transcribed into cDNA, and cell-associated (CA) unspliced (US) and multiply spliced (MS) HIV RNA was measured by qPCR. Additionally, cell culture supernatants were collected for the analysis of supernatant (SN) HIV RNA by qPCR. CA-US (**B,E,H,K**), CA-MS (**C,F,I,L**) and SN (**D,G,J**) HIV RNA is shown following stimulation with DMSO, PMA+PHA or romidepsin (**B,C,D**) or treated with increasing concentrations of IFNα (**E,F,G**), anti-CD3/CD28+IL-7+IL-2 (αCD3/CD28) with or without 100 U/mL IFNα, (**H,I,J**) or treated with 100 U/mL IFNα, IFNβ or IFNω (**K,L**). Black lines indicate median values and dots represent individual donors (n = 6–7 donors). *p<0.05, **p<0.01, ***p<0.001, ns = not significant, as determined by Wilcoxon matched pairs signed rank test.

To verify these results *ex vivo*, total CD4^+^ T cells isolated from PBMC obtained via leukapheresis from people living with HIV (PLWH) on suppressive ART (n = 7) were cultured in the presence of an integrase inhibitor and treated with IFNα or other commonly used latency reversing agents (LRAs). The demographic and clinical details are summarized in Table 1. After 3 days, cells were harvested, RNA isolated and reverse transcribed into cDNA. Cell-associated (CA) unspliced (US) and multiply spliced (MS) HIV RNA was measured by qPCR as a measure of HIV transcription (Fig 4A). Additionally, HIV RNA in cell culture supernatant (SN HIV RNA) was measured by qPCR as a measure of virus production. Treatment of the cells with PMA + PHA compared to the DMSO control treated cells increased the expression of CA-US, CA-MS and SN HIV RNA as expected (MFC CA-US 3.8, p = 0.063, n = 5; MFC CA-MS 78.3, p = 0.031, n = 6; MFC SN HIV RNA 38.0; p = 0.0313, n = 6) (Fig 4B–4D). The HDAC inhibitor romidepsin increased the expression of CA-US, CA-MS but not SN HIV RNA (MFC CA-US 8.8, p = 0.031; MFC CA-MS 31.8, p = 0.031, MFC SN HIV RNA 2.6; p = 0.0625, n = 6) (Fig 4B–4D). Treatment of these cells with increasing concentrations of IFNα *ex vivo* also increased CA-US RNA (MFC at 10 U/mL CA-US 2.4, p = 0.047; MFC at 100 U/mL CA-US 3.5, p = 0.016; MFC at 1000 U/mL CA-US 2.9, p = 0.016, n = 7) (Fig 4E) but there was no significant increase in CA-MS RNA or SN RNA (Fig 4F and 4G).

**Table 1:** Demographics of PLWH who provided blood for the *ex vivo* experiments. Triumeq = abacavir / lamivudine / dolutegravir; Truvada = tenofovir disoproxil fumarate / emtricitabine; NVP = Nevirapine; RAL = Raltegravir; ATV = Atazanavir; TDF = Tenofovir = tenofovir disoproxil fumarate; 3TC = lamivudine; DRV = Darunavir; RTV = Ritonovir; DRV/r–Darunavir/ritonavir; Genvoya = tenofovir alafenamide / emtricitabine / elvitegravir / cobicistat.

Participant	Age (years)	Sex (M or F)	Current CD4+ T cells (cells/uL)	Viral load, (copies/mL)	ART regimen	Duration ART (years)
1	65	M	767	<20	Triumeq	18.8
2	59	M	280	<20	Truvada / NVP	20.5
3	51	M	372	<20	RAL / ATV / TDF / 3TC	19.0
4	57	M	624	<20	RAL / DRV	14.7
5	58	M	520	<20	3TC / DRV/r / NVP	11.3
6	63	M	463	<20	ATV / Truvada	13.2
7	49	M	474	<20	Genvoya	8.5
Median (IQR)	58 (54–61)		474 (417.5–572)	<20		14.7 (12.3–18.9)

Anti-CD3/CD28+IL-7+IL-2 alone significantly induced CA-US RNA (MFC 4.7, p = 0.031, n = 6), and also when anti-CD3/CD28+IL-7+IL-2 was combined with IFNα (MFC 6.8, p = 0.031, n = 6, Fig 4H). CA-MS RNA increased significantly following the addition of anti-CD3/CD28+IL-7+IL-2 alone (MFC 20.0, p = 0.016, n = 7) or with the addition of IFNα and anti-CD3/CD28+IL-7+IL-2 (MFC 33.1, p = 0.016, n = 7, Fig 4I). There was no significant change in SN HIV RNA after treatment with anti-CD3/CD28+IL-7+IL-2 alone or with IFNα and anti-CD3/CD28+IL-7+IL-2 (Fig 4J). *Ex vivo* treatment of the HIV infected cells with IFNβ or IFNω had no effect on CA-US or CA-MS RNA (Fig 4K and 4L). Together, these findings show that IFNα but not IFNβ or IFNω can lead to the initiation of HIV transcription in latently infected cells both *in vitro* and *ex vivo*.

### Type I IFNs enhance expression of cell activation markers

We hypothesized that type I IFN-induced HIV expression *in vitro* and *ex vivo* may be through T cell activation. To test this, CD4^+^ T cells were cultured in the absence or presence of increasing concentrations of IFNα, β and ω (10–10,000 U/mL). After 3 days, the cells were analyzed by flow cytometry for expression of the cell surface activation markers CD69, CD25 and HLA-DR. Following IFN treatment, no change in cell viability was observed (S4A Fig). The frequency of cells expressing the early cellular activation markers CD69 and CD25 increased with IFNα, β and ω. (S4B and S4C Fig). The frequency of HLA-DR^+^ cells increased with high concentrations (1,000 and 10,000 U/mL) of IFNα and β but not with ω (S5D Fig). To verify these results *ex vivo* we analyzed the expression of these activation markers on the IFN treated CD4^+^ T cells isolated from PBMC from PLWH on ART. Following IFNα treatment, the frequency of cells expressing CD69 but not CD25 nor HLA-DR increased significantly (S4E–S4H Fig). Given all tested type I IFNs modestly activated CD4^+^ T cells, this pathway would not explain why IFNα, and not IFNβ and ω, could reverse latency.

### IFNα, IFNβ and IFNω induced phosphorylation of STAT1, 3 and 5

As IFNα, IFNβ and IFNω enhanced cell activation but only IFNα induced HIV transcription, we determined whether there was a difference in signaling downstream of the IFN receptor. All type I IFNs signal through IFNAR, transducing signals via the Janus kinase/Signal Transducer And Activator Of Transcription (JAK/STAT) and other pathways to regulate the expression of a large repertoire of ISG [46]. Conventional STAT signaling includes homodimers composed of phosphorylated (p)STAT1 and heterodimers of pSTAT1/pSTAT2 [47]. Non-conventional signaling can include dimerization of pSTAT3 and pSTAT5 proteins [48, 49], with pSTAT5 being reported to (i) induce HIV transcription via binding to the HIV LTR promoter [50], (ii) increase HIV production from primary CD4^+^ T cells [51] and (iii) to be involved in HIV latency reversal [52].

**Fig 5:**
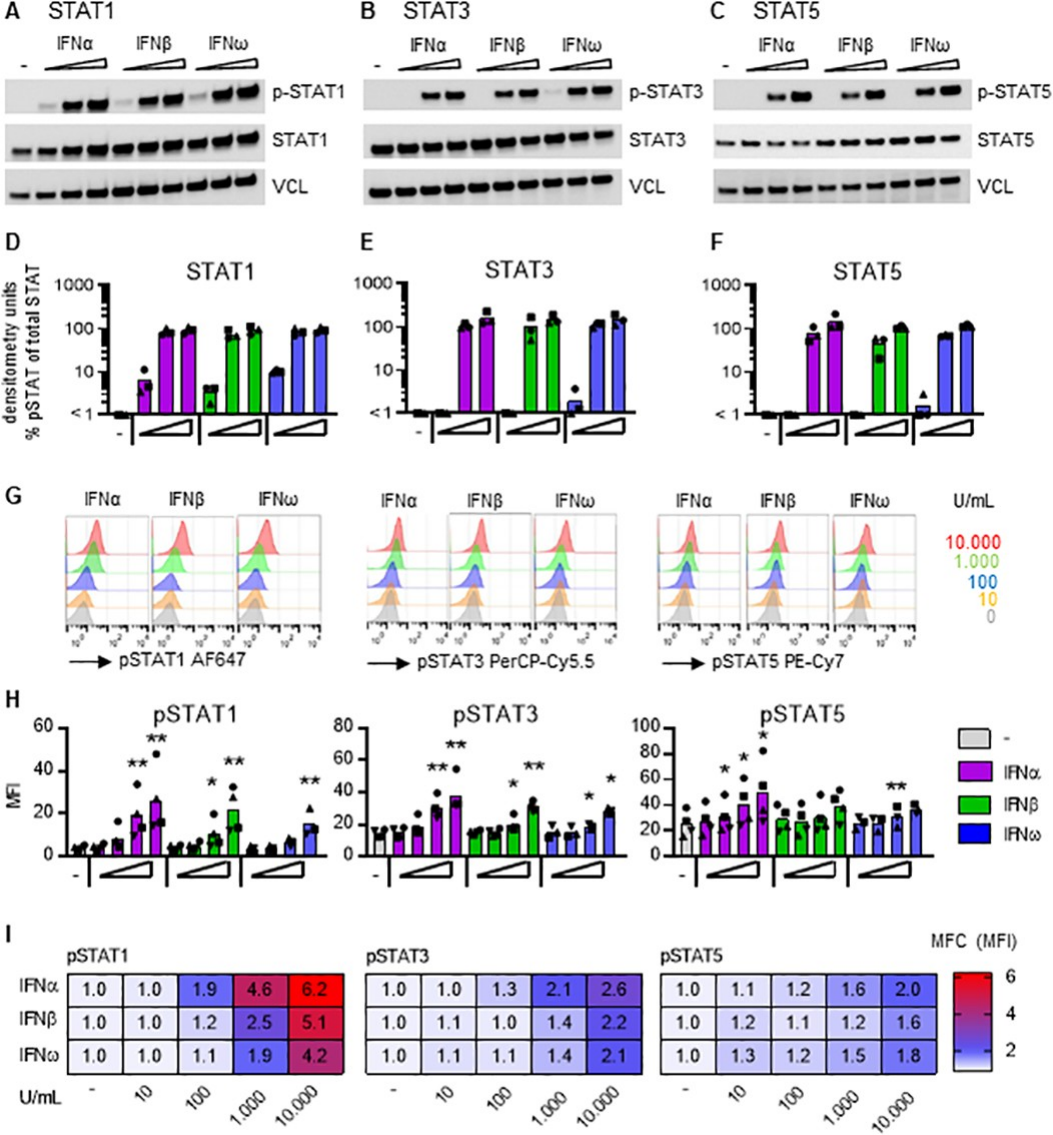
IFN-induced phosphorylation of STAT. Total CD4^+^ T cells, isolated from blood donors not living with HIV, were treated for 10 minutes with increasing (0; 100; 1,000; 10,000 U/mL) concentrations of IFNα, IFNβ or IFNω, and phosphorylation of **A:** STAT1, **B:** STAT3 and **C:** STAT5 was detected by Western Blot analysis of cell lysates. Figure shows a representative donor from a total of 3 individual donors that were obtained during 2 independently performed experiments. P-STAT = phosphorylated STAT, STAT = total (unphosphorylated) STAT, VCL = vinculin that is used as loading control. Graphs depict the densitometry analysis of the immunoblot band intensity and show relative STAT phosphorylation as compared to total STAT; **D:** STAT1, **E:** STAT3, **F:** STAT5. **G-I:** Phosphorylation of (p)STAT1, STAT3 and STAT5 was measured in total CD4^+^ T cells, isolated from HIV-uninfected blood donors, by flow cytometry after 15 minute treatment with increasing (0–10,000 U/mL) concentrations of IFNα, IFNβ or IFNω, as indicated. **G:** Representative histograms of flow cytometry analysis showing the IFN-induced change in mean fluorescent intensity (MFI) of the different STAT proteins. **H:** Graphs showing the IFN-induced changes in pSTAT MFI, bars represent mean values and each symbol represents an individual donor (n = 3–4). **I:** Heat map shows the mean fold change (MFC) from 3–4 donors of the mean fluorescent intensity (MFI) of the treated condition (shown in H) relative to the untreated condition. *p<0.05, ***p<0.001, as determined with paired student T test. MFI statistics were done on log-transformed data. Fold change calculations used non-transformed raw data.

To evaluate STAT phosphorylation, primary human CD4^+^ T cells were stimulated with increasing concentrations (10–10,000 U/mL) of IFNα, IFNβ or IFNω and phosphorylation of STAT1, 3 and 5 was detected using Western Blot analysis (Fig 5A–5F, S5A–S5F Fig) and flow cytometry (Fig 5G–5I). All 3 IFNs induced phosphorylation with IFNα being the most potent inducer of phosphorylation compared to IFNβ, which in turn was more potent than IFNω (Fig 5I). IFNα-induced phosphorylation of STAT1 was higher than STAT3, which in turn was higher than STAT5. No significant differences in the kinetics of STAT phosphorylation was observed at 15 and 30 minutes of IFN treatment but at 5 minutes, phosphorylation of STAT1 and STAT3 was significantly higher with IFNα than with IFNω treatment (S7 Fig).

Type I IFN has been reported to activate nuclear factor kappa-light-chain-enhancer of activated B cells (NFκB) [53, 54] and since the core promoter region of HIV contains two NFκB-binding sites that are crucial for HIV transcription and replication [55, 56], we investigated if type I IFN could activate NFκB in primary CD4^+^ T cells by Western Blot analysis. Treatment of CD4^+^ T cells with 10,000 U/mL IFNα, IFNβ or IFNω did not induce activation of the canonical NFκB pathway, as measured by the phosphorylation of p65 (RelA) and degradation of nuclear factor of kappa light polypeptide gene enhancer in B cells inhibitor (IκBα) (S6A–S6G Fig), except for donor #93 where IκBα appeared to be slightly degraded after IFNα treatment compared to the untreated control. Additionally, no activation of the non-canonical NFκB pathway was detected, as measured by the processing of the p100 unit into the mature subunit p52 (S5H Fig).

Taken together, these data demonstrate that activation of HIV transcription via type I IFNs is likely induced via STAT signaling rather than NFκB signaling. All three type I IFNs tested led to the phosphorylation of STAT1, 3 and 5 in primary CD4^+^ T cells but the maximum effects were observed with IFNα.

## Discussion

Interferons have well known antiviral properties and can directly inhibit HIV replication in activated CD4^+^ T cells. Here we dissected the complex effects that interferons have on latent HIV infection. Using an *in vitro* model of HIV latency with co-cultures of resting CD4^+^ T cells and DCs, we demonstrated that the inhibitory effects of pDCs on the establishment of HIV latency was mediated by a soluble factor. Blocking type I IFN signaling in infected co-cultures of CD4^+^ T cells with pDCs reversed the inhibitory effect of pDCs and the addition of IFNα to infected co-cultures of mDCs and CD4^+^ T cells could mimic the inhibitory effects of the pDCs. Once HIV latency was established, we demonstrated in both an *in vitro* model and *ex vivo* using cells from PLWH on ART, that IFNα can also reverse HIV latency through activation of HIV transcription, potentially mediated through phosphorylation of STAT5. Together these data show multiple steps where IFNs can modulate HIV latency, including a clear role of IFNα as a latency reversing agent.

We found that all type I IFNs inhibited the establishment of latent HIV infection. IFNα inhibited infection prior to integration which was not observed with IFNβ and IFNω. An intriguing aspect of the type I IFN receptor is that it can discriminate between the different type I IFNs and elicit different biological responses based on which ligand is bound [57, 58].

Using our *in vitro* model, we showed that IFNα activated HIV expression with an increase in EGFP in vitro and in CD4^+^ T cells from PLWH on ART, we observed an increase in US but not MS RNA or virion production. IFNα also had the greatest effects on activating the phosphorylation of STAT1, 3 and 5 in total CD4^+^ T cells. We observed no IFN-induced effect on the NFκB pathway and although other type I IFN-induced signal transduction pathways cannot be excluded (reviewed in [59]), STAT5 is a known activator of the HIV LTR [50] and therefore is a plausible pathway for the action of IFNα to mediate latency reversal. Studies from others have shown that HIV production is enhanced in primary CD4^+^ T cells following STAT5 activation [51] and when STAT5 activation is increased through inhibition of degradation, activation of latent HIV occurred [52].

We show that IFNα can activate latent HIV *in vitro* and *ex vivo*. In our *in vitro* model, this resulted in expression of protein (as measured by EGFP expression), while *ex vivo*, only an increase in US RNA was observed and there was no increase in MS RNA or virion release (Fig 4G). These data are similar to the effects of other LRAs, such as histone deacetylase inhibitors (HDACi) or the bromodomain inhibitor JQ1 on patient derived cells *ex vivo*, where the LRAs induced initiation of virus transcription but there were blocks to virus elongation, splicing and virion release in the absence of full T cell activation [60].

Interestingly, the administration of a TLR7 agonist, which increases multiple interferon stimulated genes and plasma levels of IFNα *in vivo* [26] and can increase virus production from latently infected cells *ex vivo* via production of IFNα [23], has a highly variable effect on virus production *in vivo*. In one study of SIV-infected rhesus macaques on ART, TLR7 agonists induced intermittent viral blips [22] but in other studies of either SIV or SIV with an HIV envelope (SHIV), no change in SIV RNA in plasma was observed [24–26]. In a recent clinical trial of the TLR7 agonist GS-9620 in PLWH on ART, there was no change in plasma HIV RNA observed. The variable effects of TLR7 agonists *in vivo* on latency reversal may be explained by the frequency and transcriptional activity of infected cells that persist on ART [26]. This could also explain some of the differences we found in the response to IFNα using our *in vitro* model where the frequency of infected cells is higher (roughly 1 in 100 to 1 in 1000 cells) and where protein was produced, compared to CD4^+^ T cells from PLWH on ART where the frequency of infected cells is low (roughly 1 in 1000 to 1 in a million cells [61]) where there was induction of US RNA only. Either way, our data clearly show that IFNα can perturb latency using both an *in vitro* and *ex vivo* model.

Of the published clinical trials of IFNα in PLWH individuals on ART [15, 16], only one study examined the changes in CA HIV RNA [19]. In this study of IFNα + ribavirin (RBV) in HCV/HIV co-infected individuals on ART, a decline in CA HIV RNA was observed, and there was no change in integrated HIV DNA [19]. Some potential explanations for these findings include the time of sample collection for analysis, i.e. 28 days post-treatment, and the inclusion of RBV in the treatment. One other study examined the effect of IFNα *in vitro* and found there to be no change in CA HIV RNA [17]. A possible explanation here could be that PBMC were treated with IFNα whereas in our study the CD4^+^ T cells are isolated prior to IFNα treatment. Another explanation is the type of IFNα used. Type I IFNα consists of 12 different subtypes that are described to have differential effects on HIV and SIV replication [62]. Here, we used a universal type I IFNα and did not test each subtype individually, while others have used specific subtypes of IFN e.g. IFNα-2a was used in the study by Sun et al. [17]. However, IFNα subtypes can differ in their antiviral and immunomodulatory effects against HIV (reviewed in [62]) and in future work an analysis of individual IFNα subtypes and their effect on HIV latency reversal might provide further mechanistic insights.

Although our *in vitro* model had some limitations, the model provides important insights into the establishment of latency that cannot be determined using patient derived cells or animal models. In our model, cells initially expressing EGFP were defined as productively infected cells and cells that did not express EGFP after sorting, were assumed to contain latently infected cells with inducible virus. Interestingly, IFN did not have a major effect on inhibiting productive infection in this model. This could have several explanations. First, IFN can strongly inhibit virus particle release but virus spread as measured by Gag-expressing cells is less potently inhibited [63]. Our measure of productive infection was the induced expression of Nef-EGFP but in the context of IFN induced restriction factors this may underestimate the effect of IFN as expression of Nef-EGFP occurs before virus assembly and budding, the site of action of some interferon induced restriction factors [40]. Second, cell-cell transfer of HIV likely occurs in this model and IFN has less effect on this mode of infection compared to infection by cell-free virions [63]. However, one very clear finding from this model is a far greater effect of IFN inhibiting latent infection, compared to productive infection. Specifically, with 1000 U/ml of IFNα, productive infection is inhibited by 60% and latent infection is inhibited by 87%. With 100 U/ml, productive infection is inhibited by 25% and latent infection by 62% (see Fig 2B). We hypothesize that the path to productive and latent infection are different. This hypothesis is further supported by the results of IFNβ and IFNω in Fig 2B, where we find that these two IFNs, which bind the same receptor as IFNα, show prominent inhibition of latent infection but appear to have no effect on productive infection. This indicates that the molecular mechanism downstream of IFN-receptor signaling can differ upon binding of different type I IFNs to the same receptor. This mechanism of differential signaling by different IFNs is well described [64].

In conclusion, we show here that type I IFNs, and specifically IFNα, has a marked effect on both inhibiting the establishment and also reversal of HIV latency. In addition to the direct effects of IFN on HIV latency, IFN has many other favorable functions that enhance clearance of infected cells, through activation of multiple innate immune pathways. The administration of IFNα or the use of interventions that modify pathways that enhance IFNα production, should be further explored as a strategy to eliminate latently infected cells.

## Materials and methods

### Ethics approval

The use of blood samples from HIV negative donors for this study was approved by the Human Research and Ethics Committees from the Alfred Hospital (HREC156/11), Monash University (CF11/1888) and the University of Melbourne (1443071). Adult donors were recruited by the Red Cross Blood Transfusion Service and all provided written informed consent for the use of their blood products for the research. The use of blood samples from adult HIV positive donors was approved by the Alfred Hospital (HREC214/15) for the study entitled *Large volume peripheral blood mononuclear cells (PBMC) collection by leukapheresis to define HIV persistence in HIV-infected adults*.

### Cell lines

HEK 293T (obtained from Prof. P. Gorry, Burnet Institute, Australia) and TZM-bl (obtained from Prof. G. Tachedjian, Burnet Institute, Australia) cell lines were grown as a monolayer in Dulbecco’s minimal essential medium (DMEM, Gibco) supplemented with 10% (v/v) heat-inactivated fetal calf serum (FCS) (Bovogene, Keilor East, Australia), 100 U/mL penicillin, 100 μg/mL streptomycin and 292 μg/mL glutamine (Gibco) at 37°C and 5% CO_2_. The hybridoma cell lines, OKT8 (ATCC Cat# CRL-8014), OKM1 (ATCC Cat# CRL-8026), 3G8 (A/Prof. A. Jaworowski, Burnet Institute, Australia), 2–06 (A/Prof. K. Shortman, WEHI, Australia), FMC63 (Flinders Medical Centre, Adelaide, Australia), FMC17 (Flinders Medical Centre, Adelaide, Australia), GlyA (A/Prof. K. Shortman, WEHI, Australia) and OKT3 (ATCC Cat# CRL-8026), were cultured in RPMI 1640 medium (Life Technologies) supplemented with 10% (v/v) heat inactivated FCS, 100 U/mL penicillin, 100 μg/mL streptomycin and 292 μg/mL glutamine (RF10) at 37°C and 5% CO_2_.

### Primary cells

Primary cells were cultured in RPMI 1640 medium (Life Technologies) supplemented with 10% (v/v) heat inactivated FCS, 100 U/mL penicillin, 100 μg/mL streptomycin and 292 μg/mL glutamine (RF10) at 37°C and 5% CO_2_.

PBMC were isolated by Ficoll-Paque density gradient centrifugation (GE Healthcare, Chalfont St. Giles, UK) from buffy coats obtained from the Australian Red Cross Blood Service (Melbourne, Australia). Resting CD4^+^ T cells were negatively selected using magnetic cell sorting with an in-house cocktail of antibodies to CD8 (clone OKT8), CD11b (OKM1), CD16 (3G8), HLA-DR (2–06), CD19 (FMC63), CD14 (FMC17) and CD69 (L78, BD Pharmingen), goat-anti-mouse IgG magnetic beads (Miltenyi Biotech) and an AutoMACS Pro (Miltenyi Biotech) as previously described [30, 65, 66]. Sorted cells were routinely negative for CD69, CD25 and HLA-DR. Total CD4^+^ T cells were isolated similarly to the resting CD4^+^ T cells with the exception that antibodies to HLA-DR and CD69 were excluded from the hybridoma cocktail. DC were isolated from blood as previously described [30, 67]. Briefly, DC were enriched using magnetic bead depletion and antibodies to CD3, CD11b and CD19. Enriched cells were then sorted using a FACSAria (BD Biosciences) or MoFlo Astrios (Beckman Coulter) Cell sorters (BD Biosciences) to obtain HLA-DR^+^ CD11c^+^ mDC or HLA-DR^+^ CD123^+^ pDC with mouse-anti-human(m-a-h)HLA-DR-APC-Cy7 (clone L243), m-a-hCD11c-V450 (b-ly6) and m-a-hCD123-PE (9F5) (BD Biosciences).

### *In vitro* DC-latency model

Resting CD4^+^ T cells (≥96 purity based on CD3^+^ and CD4^+^ expression with m-a-hCD3-PE (HIT3a) and m-a-hCD4-FITC (M5E2, BD Biosciences)) were labelled with proliferation dye eFluor670 (eBiosciences), per the manufacturer’s instructions. Labelled T cells were cultured with or without syngeneic DC (DC: T cell ratio of 1:10) with 20 ng/mL SEB (Sigma) and 10 U/mL recombinant human (r-h)IL-2 (Roche Diagnostics) for 24 hrs. Cells were infected with full length Nef-competent EGFP-reporter virus for 2–3 hrs., excess virus was washed away and cells were cultured for 5 days in media supplemented with 20 ng/mL SEB and 10 U/mL IL-2.

For transwell experiments, eFluor670-labelled T cells were cultured with mDC in the bottom chamber and pDC in the top chamber of a 0.3 μm cell culture insert (BD, Franklin Lakes, NJ). To irradiate DC, cells were exposed to 3000 rad (30 Gy), using a Gammacell 1000 Elite Cesium Source Irradiator (Nordion International Inc) or a Phoenix Cobalt-60 irradiator (Best Theratronic). In some experiments, soluble r-hIFN (universal type I IFNalpha, R&D Systems; r-hIFNbeta1a, r-hIFN-omega, h-IL-29/IFN-lambda 1 and h-IL-28B/IFN-lambda all from PBL Interferon source; IFNε was made in house as described previously [42], antibodies neutralizing soluble IFN, antibodies blocking the type I IFN receptor (IFN-alpha clone MMHA-1 and MMHA-2, IFN-beta MMHB-2, the type I IFN receptor; anti-Human Interferon Alpha/Beta Receptor Chain 2 (MMHAR-2); all from PBL Interferon source) or isotype controls (purified mouse IgG1 (MOPC-21) and IgG2a (MOPC-173) from BioLegend) were added at indicated concentrations for the r-hIFNs and 10 μg/mL for the antibodies, at the start of the culture and subsequently re-added after the wash step that follows infection.

Productive infection was determined on day 5 post-infection by detecting EGFP^+^ cells using flow cytometry. Subsequently, the non-productively infected (EGFP^-^), CD3^+^, HLA-DR^-^ (m-a-hCD3-PB, UCHT1, m-a-hHLA-DR-BV650, G46-6, from BD Biosciences) non-proliferating (eFluor670^HI^) and proliferating (eFluor670^LO^) CD4^+^ T cells were sorted using a FACSAria, MoFlo Astrios or FACSAria Fusion Cell Sorters (BD Biosciences).

To determine latent infection, 100,000–200,000 sorted T cells were cultured in a flat bottom 96 well plate in 200 μL RF10 supplemented with integrase inhibitor L870812 (Merck, White House Station, NJ, USA) or RAL (AIDS Research and Reference Reagent Program, Division of AIDS, NIAID, NIH; or Selleck Chem), both used at a final concentration of 1 μM. Cells were cultured in the presence or absence of activation stimuli composed of 3 μg/mL plate bound m-a-hCD3 (UCHT1, BD Biosciences), 2 μg/mL soluble m-a-hCD28 (L293, BD Biosciences), 50 ng/mL r-hIL-7 (Sigma) and 10 U/mL r-hIL-2. Cells were harvested 72 hrs after stimulation and EGFP expression was quantified using a FacsCalibur or FacsCanto II (BD BioSciences). Results were analyzed using Weasel software (Walter and Elisa Hall Institute, Melbourne, Australia), and to quantify latent infection, the number of EGFP^+^ cells in the unstimulated culture (background) was subtracted from the number of EGFP^+^ cells in the stimulated culture.

In some experiments, 100 U/mL IFN was added to activate latent infection, for these experiments the IFN was added to sorted cells with and without the activation stimuli for 72 hrs.

### *Ex vivo* studies of CD4^+^ T cells from people living with HIV on ART

#### Cell cultures

Leukapheresis samples were collected from PLWH on suppressive ART at the Alfred Hospital, Melbourne, Australia, with informed consent and under institutional guidelines. PBMC were isolated by Ficoll-Paque density gradient centrifugation (GE Healthcare, Chalfont St. Giles, UK) and frozen in 50–100 million cells per aliquot in liquid nitrogen. Upon thawing total CD4^+^ T cells were isolated by negative selection (StemCell Technologies). Cells were cultured at 5 million cells per well, with the exception of cells reactivated with anti-CD3/anti-CD28+IL-7+IL-2, which were cultured at 2.5 million cells per well, in a 24-well plate in the presence of RAL (1 μM, Selleck Chem) and recombinant-human (r-h)IL-2 (Roche Diagnostics). Cells were reactivated with DMSO (1:5000, Sigma Aldrich), romidepsin (40nM, Selleck Chem), PMA/PHA (10nM, Sigma Aldrich; 10 μg/mL, Thermo Fisher Scientific), universal type I IFNα (10–10,000 U/mL, R&D Systems), recombinant-human (r-h)IFNβ1a (100 U/mL PBL Interferon source), or r-hIFNω (100 U/mL PBL Interferon source). For anti-CD3/anti-CD28+IL-7+IL-2 stimulation, cells were treated with 3 μg/mL plate bound m-a-hCD3 (UCHT1, BD Biosciences), 2 μg/mL soluble m-a-hCD28 (L293, BD Biosciences), 50 ng/mL r-hIL-7 (Sigma) and 10 U/mL r-hIL-2. After 72 hrs. cells were harvested in Trizol (Life Technologies, Carlsbad, CA).

Cell-associated RNA was extracted following the manufacturer’s protocol and the RNA was then DNase-treated with RQ1 DNase (Promega).

#### cDNA synthesis

500ng of RNA for each sample was reverse transcribed into cDNA in two step incubation, the first using 0.37 mM of each dNTP, 1.5 ug random hexamers and 0.25 ug Oligo dT_(12–18)_ in a final reaction volume of 13.5 uL and incubated for 5 minutes at 65°C followed by a 5 minute cool down on ice. The second step encompassed the addition of a 6.5 uL reaction volume to reach a total final volume of 20 uL with a final concentration of 1x First strand buffer, 5 mM DTT, 20 U RNAseOUT RNAse inhibitor and 5% (v/v) Superscript III Reverse Transcriptase (Life Technologies, Austin TX). This total mixture was incubated at 42°C for 45 minutes and 80°C for 5 minutes to inactivate the RNAseOUT.

#### Semi-nested real time qPCR

125ng of cDNA per condition was then used to perform a semi-nested real time quantitative (q) PCR assay for cell-associated, unspliced HIV RNA, as previously described [68, 69] with the following primers first round forward 5’-AACTAGGGAACCCACTGCTTAAG-3’ [70] and reverse 5'-TCTCCTTCTAGCCTCCGCTAGTC-3' [71]. Second round SYBR Green (Agilent Technologies) primers (Sigma Aldrich) forward 5’-TCTCTAGCAGTGGCGCCCGAACA-3’ [71] and reverse 5’-TCTCCTTCTAGCCTCCGCTAGTC-3’ [71]. Multiply spliced HIV RNA was also measured using a semi-nested real time quantitative (q) PCR assay using the following primers: first round forward 5'-CTTAGGCATCTCCTATGGCAGGAA-3' [71] and reverse 5’-TCAAGCGGTGGTAGCTGAAGAGG-3’. Second round SYBR Green (Agilent Technologies) primers (Sigma Aldrich) forward 5’-CTTAGGCATCTCCTATGGCAGGAA-3’ [71] and reverse 5’-TTCCTTCGGGCCTGTCGGGTCCC-3’ [71]. The specific PCR conditions for both the US and MS HIV RNA PCRs are: 10% (v/v) Amplitaq Buffer II, 2.5 mM MgCl_2_, 0.2 mM of each dNTP, 0.4 uM of each primer and 0.5% (v/v) Amplitaq Gold polymerase, in a final reaction volume of 50 uL and run at 95°10’; 15x(94° 20”; 55° 40”; 72° 40”), except that for the MS assay the PCR was run for 17 cycles. For the second round PCR, 2 uL first round PCR reaction mix was used as template together with 47% (v/v) SYBR Green mix and 0.75 uM of each primer in a final volume of 26.5 uL and run at 95° 10’; 40x(94° 20”; 55° 40”) on a Stratagene MX3000.

The limit of detection for both the US and MS qRT-PCR assays was 1 copy per well. All samples were run in quadruplicate with two no RT control wells to assess for DNA contamination.

#### Quantification of supernatant HIV RN

After the 3 day incubation, culture supernatant was harvested by centrifugation at 1000 x g for 10 minutes to remove cells and debris. 1 mL of supernatant from each condition was then run on the COBAS AmpliPrep/COBAS TaqMan HIV-1 test kit v2.0 for the quantification of HIV-1 RNA (Roche Diagnostics). Culture supernatants assessed using this method have previously been shown to be free of contaminating HIV-1 DNA [72]. The limit of quantification for this assay is 20 copies/mL so anything that was undetectable or <20 was assigned a value of 20 copies/mL.

### Virus and infection

Plasmid DNA encoding a NL4.3 backbone with an AD8 envelope and EGFP inserted 1 base downstream of the *env* open reading frame [73] followed by an IRES-Nef coding sequence, kindly provided by Yasuko Tsunetsugu-Yokota (National Institute of Infectious Diseases, Tokyo, Japan), was transiently transfected into HEK 293T cells with calcium phosphate precipitation [74]. Virus supernatant was harvested after 2 days, filtered by passage through a 0.2 μm filter, concentrated using 20% sucrose density ultracentrifugation and stored in aliquots at -80°C. Cells were infected for 2–3 hrs. at an MOI of 0.5 as determined by limiting dilution on TZM-bl using the Reed and Muench method [75], followed by a wash step to remove unbound virus and luciferase as a read-out for infection (Luciferase Assay system, Promega).

### Cell viability and cell activation assays

Total CD4^+^ T cells (100,000–200,000 cells per well) were cultured in a flat bottom 96 well plate in 200 μL RF10, in the presence or absence of 3 μg/mL plate-bound m-a-hCD3, 2 μg/mL soluble m-a-hCD28, 50 ng/mL r-hIL-7 and 10 U/mL r-hIL-2 and in the absence or presence of indicated concentration r-hIFN. Cells and supernatant were harvested 72 hrs. after stimulation. Supernatant was stored at -80°C thawed and IFNγ was measured with the Human IFN-gamma ELISA MAX Deluxe kit (Biolegend) according to the manufacturer’s instructions. Cells were incubated with live/dead stain (LIVE/DEAD Fixable Aqua Dead Cell Stain Kit, for 405 nm excitation, Thermo Fisher), m-a-hCD69-FITC (L78, BD Bioscience), m-a-hCD25-PE (2A3, BD Bioscience) and m-a-hHLA-DR-APC-Cy7, and expression was quantified using a BD LSR II (BD Biosciences). Results were analyzed using Weasel software. BD CompBeads and fluorescent-minus-one (FMO) controls were used for instrument and gating setup.

### STAT phosphorylation by flow cytometry

Phosphorylation of STAT1, 3 and 5 was determined as previously described [76, 77]. Total CD4^+^ T cells were thawed, rested overnight in RF10, plated at 300,000 cells per condition in RF10 in a U bottom 96 well plate and rested for 90 min followed by a 15 min, or otherwise indicated, stimulation with r-hIFN (1–10,000 U/mL as indicated) at 37°C. As positive control for STAT phosphorylation, cells were incubated for 15 min with 20 U/mL r-hIL-2, 50 ng/mL IL-6 (InvivoGen) and 10,000 U/mL IFNα. Cells were fixed with formaldehyde (1.6% final concertation), permeabilized with ice-cold methanol and incubated with m-a-hpSTAT1-AF647 (clone 4a pY701), m-a-hSTAT3-PerCP-Cy5.5 (clone 4/P-STAT3, pY705) and m-a-hSTAT5-PE-Cy7 (clone 47, pY694). Expression was quantified using a BD LSR II (BD Biosciences) and results were analyzed using Weasel software. BD CompBeads and fluorescent-minus-one (FMO) controls were used for instrument and gating setup.

### Western Blot analysis

PBMC, obtained from HIV-negative donors, were thawed and total CD4^+^ T cells were isolated using negative magnetic selection (EasySep human CD4^+^ T cell enrichment kit, Stemcell Technologies, cat#19052). Cells were rested overnight in RF10 + r-hIL-2 (2U/mL), 1–1.2 million cells per condition were added into eppendorph tubes and rested for 90 min followed by a 10 min stimulation with r-hIFN (100–10,000 U/mL, as indicated) or r-hTNFα (100 ng/mL, PeproTech). As positive control for p50 detection, lysates of THP-1 cells (ATCC: TIB-202) treated with HT-DNA were used. 50.000 cells were seeded in 96-well plates and treated for 4 hrs. with 2 ug/mL HT-DNA (Deoxyribonucleic acid sodium salt from herring testes Sigma-Aldrich D6898).

Cells were washed with ice cold PBS and lysed in 50 uL Ripa buffer (Thermofisher Scientific) supplemented with Pierce protease and phosphatase inhibitors (Thermofisher Scientific, A32961), Complete Ultra protease inhibitor (Roche, 05892791001), Sodium fluoride (Avantar) and Benzonase Nuclease (Sigma-Aldrich), for 15 minutes on ice and stored at -20°C. Samples were thawed on ice, diluted 1:1 (v/v) with Laemmli sample buffer (Sigma-Aldrich, S3401), incubated at 95°C for 4 min, cooled on ice for min 5 min and 15 uL was loaded together with BlueStar PLUS Prestained Protein Marker (Nippon Genetics Europe, MWP04) onto a 4–20% Criterion TGX Precast Midi Protein Gel (26 well Bio-Rad, 5671095) in Nu PAGE MOPS SDS running buffer (Thermo Scientific NP0001). Proteins were transferred onto Trans-Blot Turbo Midi PVDF Transfer membrane (Bio-Rad, 170–4157) using the Trans-Blot Turbo Transfer System (Bio-Rad). Membranes were washed using Tris-buffered saline (Fisher Scientific) supplemented with 0,05% (v/v) Tween 20 (Sigma-Aldrich) (TBS-T),blocked for 1 hr. at RT in 5% Skim Milk Powder (Sigma-Aldrich) in TBS-T, washed with TBS-T, incubated o/n at 4°C with primary antibody diluted 1:1000 in 5% Bovine Serum Albumin Fraction V (Roche 10735086001) in TBS-T. The following morning, membranes were washed with TBS-T, incubated for 1 hr. with secondary antibody diluted 1:7500 in 5% Skim Milk, washed, and proteins were visualized using Clarity Western ECL Substrate (Bio-Rad, 170560) and an ImageQuant LAS 4000 mini. Membranes were washed with TBS-T, stripped for 30 sec using Restore PLUS Western Blot Stripping buffer (Thermo Fisher Scientific, 46430), washed with TBS-T, blocked for 1 hr. with 5% Skim Milk, washed with TBS-T, and incubated o/n at 4°C with another primary antibody.

To detect phosphorylated p65, rabbit-anti-human (r-a-h)p-p65 (Ser536, clone 93H1, 65 kDa, Cell Signaling Technology, cat#3033) was used. The same membrane was used to detect total p65 (r-a-hp65, clone D14E12, 65kDa, Cell Signaling Technology, cat#8242) after which the membrane was cut into two to detect IκBα (mouse-anti-human (m-a-h)-IκBα, clone L35A5, 40kDa, Cell Signaling Technology, cat#4814) and vinculin as loading control (m-a-hVCL, clone hVIN-1, 116 kDa, Sigma-Aldrich, cat#V9131).

To detect p100/p52 (NFkB2), r-a-hNFkB2 (clone 18D10, 112 and 52 kDa, Cell Signaling Technology, cat#3017) was used followed by vinculin detection as loading control.

To detect phosphorylated STAT1, r-a-hpSTAT1 (Tyr701, clone D4A7, 85 kDa, Cell Signaling Technology, cat#7649) was used. The same membrane was used to detect total STAT1 (r-a-hSTAT1, clone D1K9Y, 85 kDa, Cell Signaling Technology, cat#14994) followed by vinculin detection as loading control.

To detect phosphorylated STAT3, r-a-hpSTAT3 (Tyr705, 80 kDa, Cell Signaling Technology, cat#9131) was used. The same membrane was used to detect total STAT3 (r-a-hSTAT3, clone 79D7, 80 kDa, Cell Signaling Technology, cat#4904) followed by vinculin detection as loading control.

To detect phosphorylated STAT5, r-a-hpSTAT5 (Tyr694, clone D47E7, 90 kDa, Cell Signaling Technology, cat#4322) was used. The same membrane was used to detect total STAT5 (r-a-hSTAT3, clone D32B, 90 kDa, Cell Signaling Technology, cat#25656) followed by vinculin detection as loading control

As secondary antibodies, peroxidase donkey-anti-rabbit and donkey-anti-mouse was used (Jackson Immuno Research 711-036-152 and 715-036-150).

Densitometry analysis of the immunoblot band intensity for was performed using the Image J software.

### Isolation of RNA and DNA for quantitative real-time PCR of *in vitro* samples

For RNA extraction, cells were stored at -80°C in RNA protect Cell Reagent (Qiagen), and RNA was isolated using AllPrep DNA/RNA Mini Kit (Qiagen). RNA was reverse transcribed into cDNA using Cell Direct qRT-PCR mix (Invitrogen). qPCR was used to quantify the levels of type I and III IFN, GAPDH and virus restriction factor gene expression as described previously [8] (S8 Fig for oligonucleotides).

To quantify integrated HIV DNA, whole cell lysates were used as input for a semi-nested real time PCR using primers for Alu repeats and the HIV LTR, as described previously [45] (S9 Fig for oligonucleotides). The specific PCR conditions are: 10% (v/v) Taq Buffer (Invitrogen), 3 mM MgCl_2_, 0.2 mM of each dNTP, 0.3 uM of each Alu primer, 0.15 uM of the HIV LTR primer and 1% (v/v) Taq polymerase (Invitrogen), in a final reaction volume of 50 uL and run at 95°8’; 12x(95° 1’; 55° 1’; 72° 10’). For the second round PCR, 0.5 uL first round PCR reaction mix was used as template together with 50% (v/v) 2x Brilliant master mix III, 1 uM of each primer and 0.125 uM of probe in a final volume of 20 uL and run at 95° 3’; 40x(95° 5”; 60° 20”) on a Stratagene MX3000. See key resources table for the oligonucleotides used. All samples were run in triplicate with an LTR primer only control to assess unintegrated DNA detection.

Alu PCR results were normalized for total input DNA as determined by real-time PCR for the CCR5 gene [78]. The specific conditions for the CCR5 PCR are: 50% (v/v) SYBR Green mix and 0.8 uM of each primer in a final volume of 25 uL and run at 95° 8’; 45x(94° 20”; 58,5° 40”; 72° 40”)72° 10’ on a Stratagene MX3000. See key resources table for the oligonucleotides used and all samples were run in duplicates.

### Standard curves for qPCR

The amounts of the CCR5 gene copies, integrated HIV DNA, CA-US and MS HIV RNA were determined by using serial dilutions of standard curves run in parallel with the samples of interest.

The standard for the CCR5 assay was generated by pooling PBMC from three different donors. Cells were counted manually in fivefold and brought to a final concentration of 40*10^6^ cells/mL. Cells were lysed using Proteinase K (0.8 mg/mL final concentration added fresh to premade lysis buffer containing 1 mM Tris pH 8, 1 mM EDTA, 0,002% (v/v) Triton 100, 0.002% (v/v) SDS), and incubation at 56°C for 1 hour followed by a Proteinase K inactivation step at 95°C for 10 minutes. Cell lysates were stored in aliquots at -80°C.

The standards for the integrated HIV DNA assays were generated following the same procedure as the CCR5 standards, except that the ACH-2 cell line was used (NIH AIDS Reagent Program, Division of AIDS, NIAID, NIH: Dr. Thomas Folks). ACH-2 cells are a human T cell line with each cell carrying one latent proviral copy of HIV (LAV strain). Cells were brought to a concentration to 4*10^6^ cells/mL, lysed and stored in aliquots at -80°C.

The standards for the RNA assays were synthetic runoff transcripts generated from the linearized plasmids SP5-Δ33-NL4.3 (digested with *Spe I* for the US RNA assay) and Sp5-Δ33-Tat2-PA (digested with *Cla I* for the MS RNA assay). The lengths of the synthetic transcripts were 1053 nucleotides (nt) for pGAG2-A5 and 1867 nt for Sp5-Δ33-Tat2-PA. The concentrations of RNA standards were determined by nanodrop and purity was confirmed by Northern Blot analysis. The standards were frozen in aliquots at a high concentration to prevent degradation (4*10^10^ copies/uL) and stored at -80°C until use.

### Quantification and statistical analysis

Differences between experimental conditions were analyzed using Wilcoxon matched pairs signed rank test (n > 5) or paired student T test (n ≤ 5) on log-transformed data with GraphPad Prism (Version 6). P-values ≤0.05 were considered significant: *p<0.05, **p<0.01, ***p<0.001, ****p<0.0001. Details can be found in the figure legend of each figure.

## Supporting information

S1 Fig Infection with HIV enhances expression of IFNα, IFNβ, IFNω and IFNλ2 mRNA in co-cultures of T cells with pDC but not with mDC. **A:** Resting CD4^+^ T cells were co-cultured with pDC in the presence of antibodies blocking IFNAR and antibodies neutralizing soluble IFNα and IFNβ (anti-IFNmix) or isotype control (iso ctrl) to quantify productive and latent infection (see Fig 1E). **B:** Resting CD4^+^ T cells were co-cultured with mDC (top) or pDC (bottom) with and without HIV infection. Cells were harvested 1 day post-infection and mRNA expression of type I and type III IFN was quantified using qPCR (n = 4). *p<0.05, as determined by paired student T test on log-transformed data.
(DOCX)

S2 Fig IFN-induced inhibition of productive infection and establishment of latent infection. Resting CD4^+^ T cells were cultured in the DC-latency model with mDC in the absence or presence of indicated IFNs and productive (purple) and latent (grey) infection in non-proliferating T cells was quantified using flow cytometry. Columns represent mean values and dots represent individual donors (n = 3–4 donors). *p<0.05, as determined by paired student T test on log-transformed data, nd = not done.
(DOCX)

S3 Fig IFNα induces expression of HIV *in vitro*. Resting CD4^+^ T cells were co-cultured with mDC in the DC-latency model. Virus expression and activation from latency was determined using sorted cells cultured in the presence of an integrase inhibitor and either left untreated, cultured with 100 U/mL IFN, activated with anti-CD3/CD28+IL-7+IL-2 (αCD3/CD28) or activated with anti-CD3/CD28+IL-7+IL-2 in the presence of 100 U/mL IFN for 3 days prior to EGFP^+^ quantification by flow cytometry. Red lines indicate median values and dots represent individual donors (n = 7–12 donors). *p<0.05, **p<0.01, ***p<0.001, ns = not significant as determined by Wilcoxon matched pairs signed rank test.
(DOCX)

S4 Fig Type I IFNs enhance expression of extracellular markers associated with T cell activation. Total CD4^+^ T cells isolated from PBMC obtained from HIV-uninfected individuals (**A-D**) or total CD4^+^ T cells isolated from PBMC obtained from HV-infected individuals on ART (**E-H**) were left untreated or treated with increasing (0–10,000 U/mL) concentrations of indicated IFN or with anti-CD3/CD28+IL-7+IL-2 (TCR-activated). After 3 days, the cells were harvested and the percentage of **A,E:** viable cells and cells expressing **B,F:** CD69, **CG:** CD25 and **D,H:** HLA-DR were measured using flow cytometry. Columns represent mean values and dots represent individual donors (n = 3–4 donors). *p<0.05, **p<0.01 as determined by paired student T test.
(DOCX)

S5 Fig Western Blot analysis of IFN-induced phosphorylation of STAT1, 3 and 5. Total CD4^+^ T cells, isolated from HIV-uninfected individuals, were treated for 10 minutes with 100, 1,000 or 10,000 U/mL of IFNα, IFNβ or IFNω or left untreated (—), as indicated, and phosphorylation of **A:** STAT1, **B:** STAT3 and **C:** STAT5 was detected by Western Blot analysis of cell lysates. Graphs depict the densitometry analysis of the immunoblot band intensity for **D:** pSTAT; **E:** total STAT and; **F:** relative pSTAT as compared to total STAT. Results from 3 individual donors that were obtained during two independently performed experiments are shown. P-STAT = phosphorylated STAT, STAT = total (unphosphorylated) STAT, VCL = vinculin that is used as loading control.
(DOCX)

S6 Fig Western Blot analysis of IFN- and TNFα-induced NFκB signaling. Total CD4^+^ T cells, isolated from HIV-uninfected individuals, were not treated (—) or treated for 10 minutes with 10,000 U/mL of IFNα, IFNβ, IFNω or 100 ng/mL TNFα. **A:** Cell lysates were analyzed by Western Blot for canonical NFκB activation by the phosphorylation of p65 (p-p65) and degradation of nuclear factor of kappa light polypeptide gene enhancer in B-cells inhibitor alpha (IκBα). Graphs depict the densitometry analysis of the immunoblot band intensity for **B:** p-p65; **C:** total p65; **D:** relative p-p65 as compared to total p65; **E:** IκBα; **F:** VCL and; **G:** relative IκBα as compared to VCL. **E:** Cell lysates were analyzed for non-canonical NFκB activation by the processing of p100 into the mature subunit p52. As a positive control for the detection of p52, lysates of the THP-1 cell line treated with herring testes (HT-)DNA were used (THP1+HT-DNA). Results from 3 individual donors that were obtained during 2 independently performed experiments are shown. P-p65 = phosphorylated p65, p65 = total (unphosphorylated) p65, VCL = vinculin that is used as loading control.
(DOCX)

S7 Fig IFN-induced phosphorylation of STAT1, 3 and 5 by flow cytometry. **A:** Total CD4^+^ T cells, isolated from HIV-uninfected individuals, were treated for 5, 15 or 30 minutes with 10,000 U/mL of indicated IFN and phosphorylation of indicated STAT was measured by flow cytometry and **B:** the mean fold change (MFC) in MFI was calculated and presented in a heatmap. Columns represent mean values and dots represent individual donors (n = 4). *p<0.05, **p<0.01 as determined by paired student T test. All MFI statistics were done on log-transformed data. Fold change calculations used raw and not log-transformed data.
(DOCX)

S8 Fig Oligonucleotides used for qPCR to quantify the levels of type I and III IFN, GAPDH and virus restriction factor gene expression. (DOCX)

S9 Fig Oligonucleotides used for qPCR to quantify the levels of HIV RNA, DNA or CCR5. (DOCX)
