# Supplementary material for: Diverse effects of interferon alpha on the establishment and reversal of HIV latency
Source: PLoS Pathog. 2020 Feb 28;16(2):e1008151. doi: 10.1371/journal.ppat.1008151 (PMC7065813; doi:10.1371/journal.ppat.1008151)
Supplement: S1 Fig — A: Resting CD4+ T cells were co-cultured with pDC in the presence of antibodies blocking IFNAR and antibodies neutralizing soluble IFNα and IFNβ (anti-IFNmix) or isotype control (iso ctrl) to quantify productive and latent infection (see Fig 1E). B: Resting CD4+ T cells were co-cultured with mDC (top) or pDC (bottom) with and without HIV infection. Cells were harvested 1 day post-infection and mRNA expression of type I and type III IFN was quantified using qPCR (n = 4). *p<0.05, as determined by paired student T test on log-transformed data. (DOCX) [file ppat.1008151.s001.docx]

**S1 Fig. Infection with HIV enhances expression of IFNα, IFNβ, IFNω and IFNλ2 mRNA in co-cultures of T cells with pDC but not with mDC. A:** Resting CD4^+^ T cells were co-cultured with pDC in the presence of antibodies blocking IFNAR and antibodies neutralizing soluble IFNα and IFNβ (anti-IFNmix) or isotype control (iso ctrl) to quantify productive and latent infection (see Fig 1E). **B:** Resting CD4^+^ T cells were co-cultured with mDC (top) or pDC (bottom) with and without HIV infection. Cells were harvested 1 day post-infection and mRNA expression of type I and type III IFN was quantified using qPCR (n=4). *p<0.05, as determined by paired student T test on log-transformed data.
