# Supplementary material for: Diverse effects of interferon alpha on the establishment and reversal of HIV latency
Source: PLoS Pathog. 2020 Feb 28;16(2):e1008151. doi: 10.1371/journal.ppat.1008151 (PMC7065813; doi:10.1371/journal.ppat.1008151)
Supplement: S2 Fig — Resting CD4+ T cells were cultured in the DC-latency model with mDC in the absence or presence of indicated IFNs and productive (purple) and latent (grey) infection in non-proliferating T cells was quantified using flow cytometry. Columns represent mean values and dots represent individual donors (n = 3–4 donors). *p<0.05, as determined by paired student T test on log-transformed data, nd = not done. (DOCX) [file ppat.1008151.s002.docx]

**S2 Fig. IFN-induced inhibition of productive infection and establishment of latent infection.** Resting CD4^+^ T cells were cultured in the DC-latency model with mDC in the absence or presence of indicated IFNs and productive (purple) and latent (grey) infection in non-proliferating T cells was quantified using flow cytometry. Columns represent mean values and dots represent individual donors (n=3-4 donors). *p<0.05, as determined by paired student T test on log-transformed data, nd=not done.
