# Supplementary material for: Diverse effects of interferon alpha on the establishment and reversal of HIV latency
Source: PLoS Pathog. 2020 Feb 28;16(2):e1008151. doi: 10.1371/journal.ppat.1008151 (PMC7065813; doi:10.1371/journal.ppat.1008151)
Supplement: S3 Fig — Resting CD4+ T cells were co-cultured with mDC in the DC-latency model. Virus expression and activation from latency was determined using sorted cells cultured in the presence of an integrase inhibitor and either left untreated, cultured with 100 U/mL IFN, activated with anti-CD3/CD28+IL-7+IL-2 (αCD3/CD28) or activated with anti-CD3/CD28+IL-7+IL-2 in the presence of 100 U/mL IFN for 3 days prior to EGFP+ quantification by flow cytometry. Red lines indicate median values and dots represent individual donors (n = 7–12 donors). *p<0.05, **p<0.01, ***p<0.001, ns = not significant as determined by Wilcoxon matched pairs signed rank test. (DOCX) [file ppat.1008151.s003.docx]

**S3 Fig. IFNα induces expression of HIV *in vitro*.**

Resting CD4^+^ T cells were co-cultured with mDC in the DC-latency model. Virus expression and activation from latency was determined using sorted cells cultured in the presence of an integrase inhibitor and either left untreated, cultured with 100 U/mL IFN, activated with anti-CD3/CD28+IL-7+IL-2 (αCD3/CD28) or activated with anti-CD3/CD28+IL-7+IL-2 in the presence of 100 U/mL IFN for 3 days prior to EGFP^+^ quantification by flow cytometry. Red lines indicate median values and dots represent individual donors (n=7-12 donors). *p<0.05, **p<0.01, ***p<0.001, ns=not significant as determined by Wilcoxon matched pairs signed rank test.
