# Supplementary material for: Diverse effects of interferon alpha on the establishment and reversal of HIV latency
Source: PLoS Pathog. 2020 Feb 28;16(2):e1008151. doi: 10.1371/journal.ppat.1008151 (PMC7065813; doi:10.1371/journal.ppat.1008151)
Supplement: S4 Fig — Total CD4+ T cells isolated from PBMC obtained from HIV-uninfected individuals (A-D) or total CD4+ T cells isolated from PBMC obtained from HV-infected individuals on ART (E-H) were left untreated or treated with increasing (0–10,000 U/mL) concentrations of indicated IFN or with anti-CD3/CD28+IL-7+IL-2 (TCR-activated). After 3 days, the cells were harvested and the percentage of A,E: viable cells and cells expressing B,F: CD69, CG: CD25 and D,H: HLA-DR were measured using flow cytometry. Columns represent mean values and dots represent individual donors (n = 3–4 donors). *p<0.05, **p<0.01 as determined by paired student T test. (DOCX) [file ppat.1008151.s004.docx]

**S4 Fig. Type I IFNs enhance expression of extracellular markers associated with T cell activation.** Total CD4^+^ T cells isolated from PBMC obtained from HIV-uninfected individuals (**A-D**) or total CD4^+^ T cells isolated from PBMC obtained from HV-infected individuals on ART (**E-H**) were left untreated or treated with increasing (0-10,000 U/mL) concentrations of indicated IFN or with anti-CD3/CD28+IL-7+IL-2 (TCR-activated). After 3 days, the cells were harvested and the percentage of **A,E:** viable cells and cells expressing **B,F:** CD69, **CG:** CD25 and **D,H:** HLA-DR were measured using flow cytometry. Columns represent mean values and dots represent individual donors (n=3-4 donors). *p<0.05, **p<0.01 as determined by paired student T test.
