# Supplementary material for: Diverse effects of interferon alpha on the establishment and reversal of HIV latency
Source: PLoS Pathog. 2020 Feb 28;16(2):e1008151. doi: 10.1371/journal.ppat.1008151 (PMC7065813; doi:10.1371/journal.ppat.1008151)
Supplement: S5 Fig — Total CD4+ T cells, isolated from HIV-uninfected individuals, were treated for 10 minutes with 100, 1,000 or 10,000 U/mL of IFNα, IFNβ or IFNω or left untreated (—), as indicated, and phosphorylation of A: STAT1, B: STAT3 and C: STAT5 was detected by Western Blot analysis of cell lysates. Graphs depict the densitometry analysis of the immunoblot band intensity for D: pSTAT; E: total STAT and; F: relative pSTAT as compared to total STAT. Results from 3 individual donors that were obtained during two independently performed experiments are shown. P-STAT = phosphorylated STAT, STAT = total (unphosphorylated) STAT, VCL = vinculin that is used as loading control. (DOCX) [file ppat.1008151.s005.docx]

**S5 Fig. Western Blot analysis of IFN-induced phosphorylation of STAT1, 3 and 5.**

Total CD4^+^ T cells, isolated from HIV-uninfected individuals, were treated for 10 minutes with 100, 1,000 or 10,000 U/mL of IFNα, IFNβ or IFNω or left untreated ( - ), as indicated, and phosphorylation of **A:** STAT1, **B:** STAT3 and **C:** STAT5 was detected by Western Blot analysis of cell lysates. Graphs depict the densitometry analysis of the immunoblot band intensity for **D:** pSTAT; **E:** total STAT and; **F:** relative pSTAT as compared to total STAT. Results from 3 individual donors that were obtained during two independently performed experiments are shown. P-STAT = phosphorylated STAT, STAT = total (unphosphorylated) STAT, VCL = vinculin that is used as loading control.
