# Supplementary material for: Diverse effects of interferon alpha on the establishment and reversal of HIV latency
Source: PLoS Pathog. 2020 Feb 28;16(2):e1008151. doi: 10.1371/journal.ppat.1008151 (PMC7065813; doi:10.1371/journal.ppat.1008151)
Supplement: S6 Fig — Total CD4+ T cells, isolated from HIV-uninfected individuals, were not treated (—) or treated for 10 minutes with 10,000 U/mL of IFNα, IFNβ, IFNω or 100 ng/mL TNFα. A: Cell lysates were analyzed by Western Blot for canonical NFκB activation by the phosphorylation of p65 (p-p65) and degradation of nuclear factor of kappa light polypeptide gene enhancer in B-cells inhibitor alpha (IκBα). Graphs depict the densitometry analysis of the immunoblot band intensity for B: p-p65; C: total p65; D: relative p-p65 as compared to total p65; E: IκBα; F: VCL and; G: relative IκBα as compared to VCL. E: Cell lysates were analyzed for non-canonical NFκB activation by the processing of p100 into the mature subunit p52. As a positive control for the detection of p52, lysates of the THP-1 cell line treated with herring testes (HT-)DNA were used (THP1+HT-DNA). Results from 3 individual donors that were obtained during 2 independently performed experiments are shown. P-p65 = phosphorylated p65, p65 = total (unphosphorylated) p65, VCL = vinculin that is used as loading control. (DOCX) [file ppat.1008151.s006.docx]

**S6 Fig. Western Blot analysis of IFN- and TNFα-induced NFκB signaling.**

Total CD4^+^ T cells, isolated from HIV-uninfected individuals, were not treated ( - ) or treated for 10 minutes with 10,000 U/mL of IFNα, IFNβ, IFNω or 100 ng/mL TNFα. **A:** Cell lysates were analyzed by Western Blot for canonical NFκB activation by the phosphorylation of p65 (p-p65) and degradation of nuclear factor of kappa light polypeptide gene enhancer in B-cells inhibitor alpha (IκBα). Graphs depict the densitometry analysis of the immunoblot band intensity for **B:** p-p65; **C:** total p65; **D:** relative p-p65 as compared to total p65; **E:** IκBα; **F:** VCL and; **G:** relative IκBα as compared to VCL. **E:** Cell lysates were analyzed for non-canonical NFκB activation by the processing of p100 into the mature subunit p52. As a positive control for the detection of p52, lysates of the THP-1 cell line treated with herring testes (HT-)DNA were used (THP1+HT-DNA). Results from 3 individual donors that were obtained during 2 independently performed experiments are shown. P-p65 = phosphorylated p65, p65 = total (unphosphorylated) p65, VCL = vinculin that is used as loading control.
