# Supplementary material for: Diverse effects of interferon alpha on the establishment and reversal of HIV latency
Source: PLoS Pathog. 2020 Feb 28;16(2):e1008151. doi: 10.1371/journal.ppat.1008151 (PMC7065813; doi:10.1371/journal.ppat.1008151)
Supplement: S7 Fig — A: Total CD4+ T cells, isolated from HIV-uninfected individuals, were treated for 5, 15 or 30 minutes with 10,000 U/mL of indicated IFN and phosphorylation of indicated STAT was measured by flow cytometry and B: the mean fold change (MFC) in MFI was calculated and presented in a heatmap. Columns represent mean values and dots represent individual donors (n = 4). *p<0.05, **p<0.01 as determined by paired student T test. All MFI statistics were done on log-transformed data. Fold change calculations used raw and not log-transformed data. (DOCX) [file ppat.1008151.s007.docx]

**S7 Fig. IFN-induced phosphorylation of STAT1, 3 and 5 by flow cytometry**

**A:** Total CD4^+^ T cells, isolated from HIV-uninfected individuals, were treated for 5, 15 or 30 minutes with 10,000 U/mL of indicated IFN and phosphorylation of indicated STAT was measured by flow cytometry and **B:** the mean fold change (MFC) in MFI was calculated and presented in a heatmap. Columns represent mean values and dots represent individual donors (n=4). *p<0.05, **p<0.01 as determined by paired student T test. All MFI statistics were done on log-transformed data. Fold change calculations used raw and not log-transformed data.
