# Supplementary material for: Diverse effects of interferon alpha on the establishment and reversal of HIV latency
Source: PLoS Pathog. 2020 Feb 28;16(2):e1008151. doi: 10.1371/journal.ppat.1008151 (PMC7065813; doi:10.1371/journal.ppat.1008151)
Supplement: S9 Fig — (DOCX) [file ppat.1008151.s009.docx]

**S9 Fig. Oligonucleotides used for qPCR to quantify the levels of HIV RNA, DNA or CCR5**
